# Profiling tumour-infiltrating immune cells in a large paediatric medulloblastoma cohort: a retrospective analysis

**DOI:** 10.1016/j.ebiom.2025.106043

**Published:** 2025-11-23

**Authors:** Mingze Chen, Xiangjun Shi, Yi Wang, Jiao Zhang, Jirong Guo, Xiuchen Guan, Yu Sun, Wenhao Wu, Chunde Li, Yongji Tian, Yunwei Ou, Tian Li, Kai Jiang, Michael D. Taylor, Xuebin Liao, Liwei Zhang, Tao Sun

**Affiliations:** a Department of Neurosurgery, Beijing Tiantan Hospital, Capital Medical University, Beijing 100070, PR China; b China National Clinical Research Centre for Neurological Diseases, Beijing Tiantan Hospital, Capital Medical University, Beijing 100070, PR China; c School of Medicine, Tsinghua University, Beijing 100084, PR China; d Texas Children’s Cancer and Hematology Centre, Houston, TX, USA; e Department of Paediatrics–Hematology/Oncology, Baylor College of Medicine, Houston, TX, USA; f Department of Orthodontics, School of Stomatology, Capital Medical University, Beijing 100070, PR China; g Department of Biomedical Engineering, The Hong Kong Polytechnic University, Hong Kong Special Administrative Region of China; h Department of Neurosurgery, Baylor College of Medicine, Houston, TX, USA; i Department of Neurosurgery, Texas Children’s Hospital, Houston, TX, USA; j Dan L Duncan Comprehensive Cancer Centre, Baylor College of Medicine, Houston, TX, USA; k The Arthur and Sonia Labatt Brain Tumour Research Centre and the Developmental and Stem Cell Biology Program, The Hospital for Sick Children, Toronto, Ontario, Canada; l Department of Surgery, University of Toronto, Toronto, Ontario, Canada; m Department of Laboratory Medicine and Pathobiology, University of Toronto, Toronto, Ontario, Canada; n Department of Medical Biophysics, University of Toronto, Toronto, Ontario, Canada; o State Key Laboratory of Molecular Oncology, School of Pharmaceutical Sciences, Tsinghua-Peking Centre for Life Science, Tsinghua University, Beijing 100084, PR China

**Keywords:** Medulloblastoma, Multiplex immunofluorescence, Tertiary lymphoid structures, Prognosis

## Abstract

**Background:**

Tumour-infiltrating immune cells exert both pro-tumour and anti-tumour effects on intracranial tumours. In this study, we investigated the prognostic value of various infiltrating immune cells in medulloblastoma (MB) within a large cohort.

**Methods:**

We employed multiplex immunofluorescent (mIF) staining of tissue microarrays to assess the densities of T cells, B cells, NK cells, macrophages, and immune checkpoints in tumour samples from 249 primary paediatric patients with primary MB. Overall survival (OS) analysis, progression-free survival (PFS), and Cox regression analyses were utilised to explore potential relationships between immune cell densities and survival outcomes. Subsequently, multivariate Cox regression was validated using an external database.

**Findings:**

Significant differences in the immune microenvironment among molecular groups of MB and their prognostic implications were observed. CD4+ T cell infiltration was positively correlated with improved OS prognosis (Hazard Ratio^low vs high^ 2.19 [95% CI 1.197–4.016]), while M2 macrophages (HR^low vs high^ 0.50 [95% CI 0.250–1.003]), NK cells (HR^low vs high^ 0.40 [95% CI 0.207–0.792]), and B cells (HR^low vs high^ 0.51 [95% CI 0.291–0.902]) were associated with poorer PFS prognosis. We detected the presence of early tertiary lymphoid structures in MB. The infiltration of B cells (HR^low vs high^ 0.50 [95% CI 0.271–0.941]) correlated with poorer OS. The prognostic significance of B cells was further corroborated in cases from external data. Additionally, multivariate Cox regression confirmed that B cells were identified as independent negative prognosticators for OS (HR^high vs low^ 2.21 [95% CI 1.341–3.651]) and PFS (HR^high vs low^ 2.15 [95% CI 1.28–3.61]).

**Interpretation:**

Our study provides a comprehensive characterisation of the spatial distribution of certain immune cells and tumour cells within the tumour core of patients with various subtypes of MB, underscoring the prognostic significance of B cells in these individuals.

**Funding:**

Beijing Municipal Public Welfare Development and Reform Pilot Project for Medical Research Institutes (PWD & RPP-MRI: JYY2018-7); 10.13039/501100001809National Natural Science Foundation of China (32300773); 10.13039/501100002858China Postdoctoral Science Foundation (2022M721864).

**Research in context**

**Evidence before this study**

Several immunotherapies have significantly improved the prognosis of intracranial malignant tumours; however, their therapeutic efficacy in paediatric medulloblastoma (MB) remains limited. MB, the most prevalent malignant brain tumour in children, is characterised by an intact blood–brain barrier and limited immune cell infiltration. Previous investigations into the immune microenvironment of MB have predominantly focused on T cells and macrophages. Some reports with small sample sizes suggest a correlation between PD-L1 expression, macrophage polarization, and survival outcomes. However, the prognostic significance of other immune cell populations, such as B cells, NK cells, and tertiary lymphoid structures, has not been systematically assessed. To date, no large-scale paediatric cohort study utilising multiplex immunofluorescence combined with external validation has comprehensively explored the immune infiltration patterns of MB and their clinical implications.

**Added value of this study**

This retrospective study included 249 paediatric patients with primary MB, making it the largest study to date on spatially resolved immune infiltration characteristics. Utilising six multiplex immunofluorescence panels, we comprehensively assessed the distribution and prognostic significance of T cell subsets, macrophages, NK cells, B cells, tertiary lymphoid structures (TLSs), and immune checkpoints. Our findings indicate that CD4+ T cell infiltration correlates with improved survival, whereas high-density infiltrations of B cells, NK cells, and M2 macrophages are associated with poorer prognosis. Notably, we report the presence of early TLSs in MB. Multivariate regression analysis demonstrated that B cells serve as independent negative prognostic factors for overall survival (OS) and progression-free survival (PFS), a finding that was further validated in an external RNA-seq cohort. Furthermore, immune checkpoint analysis indicated that CTLA-4 is linked to poorer prognosis, potentially providing a biological basis for the clinical exploration of combined immune checkpoint inhibitors.

**Implications of all the available evidence**

This study further delineates the panoramic characteristics of the MB immune microenvironment, revealing the heterogeneous roles of various immune cell subsets in disease progression. Our findings demonstrate that distinct immune cell infiltration patterns are closely associated with survival outcomes. In the multivariate analysis, B cells emerged as an independent negative prognostic indicator, underscoring their significance in risk assessment and potential therapeutic strategies. Additionally, the expression of CTLA-4 was correlated with poor prognosis, providing biological rationale for investigating combination immune checkpoint inhibition. Overall, these results not only enhance our understanding of the MB immune microenvironment but also offer essential insights for integrating immune features into prognostic assessment and the design of immune therapies.

## Introduction

Medulloblastoma (MB), an embryonal tumour of the cerebellum, is the most common malignant brain tumour in children, representing 12% of childhood brain tumours.^1^ MB is suspected to originate from various discrete neuronal stem or progenitor cell populations during early life, and has been linked to specific genetic aberrations.^2^ In the 2021 WHO classification of MB, MB is classified into molecular groups: WNT (Wingless), SHH (Sonic Hedgehog with *TP53*-mutant and *TP53*-wildtype), non-WNT/non-SHH (Group 3 and Group 4) and histological subtypes.^3^ Among the molecular subgroups, tumours in G3, particularly those characterised by MYC amplification and/or isochromosome 17q, as well as SHH with TP53 mutations, are recognised as some of the most consistently reported high-risk categories.^4^ The histopathological subtypes include classic, desmoplastic/nodular (DN), large cell/anaplastic (LC/A), and MB with extensive nodularity (MBEN).^2^^,^^3^ The LC/A subtype accounts for approximately 10% of cases and is associated with a poorer prognosis.^5^ The morphological differences are associated with specific clinical features, and molecularly defined medulloblastomas show distinct correlations with these morphological patterns.

The blood–brain barrier (BBB) serves as a physical barrier for immune cells. Traditionally, the brain has been regarded as an immune-privileged organ, shielded from immune surveillance. However, this notion has been challenged over the past decade,^6^ with increasing evidence supporting the concept of immune surveillance within the central nervous system (CNS). This process remains highly regulated, permitting only limited access for immune cells.^7^ Numerous studies have demonstrated that MB tumours are deficient in effector immune cells, likely due to the BBB’s stringent control over immune surveillance in the CNS, which restricts immune cell infiltration.^8^ Except for WNT-type tumours, most MB tumours exhibit extensive regions of an intact BBB, indicating sustained and restricted immune cell infiltration.^9^^,^^10^ A previous study reported immune cell infiltration in MB and found that PD-L1 expression correlated with poor prognosis in MB.^11^^,^^12^ However, the potential prognostic value of quantifying additional immune cell types remains uncertain. In this study we sought to investigate the prognostic values of infiltrating immune cells by multiplex immunostaining of MB in an observational cohort from our hospital. The implementation of a variety of innovative immune checkpoint-based cancer immunotherapies has dramatically altered the prognosis of several advanced cancers,^13^ yet there is limited knowledge about immune checkpoint expression and impacts in MB.

In the present study, we assessed tissue microarrays to evaluate the density of immune cell infiltration in 249 patients with MB based on six multiplex immunofluorescence panels. While T cells and macrophages the primary focus of research in the MB tumour immune microenvironment, emerging evidence indicates that B cells and NK cells may also play significant, albeit under-explored, roles.^14^^,^^15^ Recent studies have shown that NK cells in MB cell lines and xenograft models express ligands for NK activating receptors, suggesting that MB cells could be vulnerable to NK-mediated cytotoxicity.^16^ B cells exhibit various functions, both anti- and pro-tumorigenic, within the tumour microenvironment, such as producing antibodies against tumour-associated antigens (TAs) or inducing regulatory T cells. However, B cells have garnered less attention compared to the aforementioned cell types and no studies have investigated the therapeutic potential of B cells in MB.^17^^,^^18^ Therefore, including B and NK cells in our immune panel enables us to evaluate not only the canonical adaptive and innate immune responses (T cells, macrophages) but also humoral immunity, NK cytotoxicity, and immunoregulatory interactions. Our analyses ultimately combined clinical features, T cell subpopulations, macrophages, NK cells, tertiary lymphoid structures (TLSs), T cell-expressed immune checkpoints, and tumour cell-expressed immune checkpoints and assessed their potential links to patient prognosis. Additionally, we analysed the expression differences of immune cell populations in relation to molecular groups and developed an immune prognostic evaluation model for MB.

## Methods

### Tissue samples and microarray formats

**Fig. 1:**
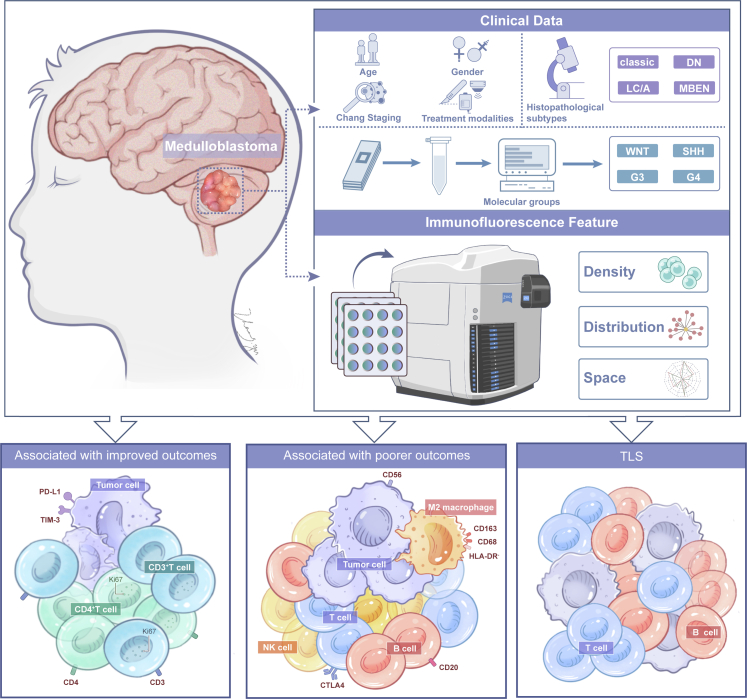
Graphical overview and summary of the major findings in this study. We collected clinical information from the patients and conducted immunofluorescence analyses to evaluate the density and spatial distance, thereby obtaining prognostic information related to MB.

To evaluate the prognostic value of immune markers, we selected a cohort of 249 patients who were registered in the Beijing Tiantan Hospital and received an initial diagnosis of medulloblastoma between 2014 and 2020 (Fig. 1). In the study design, patient sex was self-reported by the participants, and all patients included in the study were of Asian ethnicity. All patients had complete follow-up information after undergoing craniotomy and standard treatment. The tissue samples were acquired from the postoperative human brain tumour tissue centre. At the time of surgery, all patients were treatment-naive and subsequently underwent chemotherapy and/or craniospinal irradiation following the surgical procedure. Treatment was performed according to the German Society of Paediatric Oncology and Hematology (GPOH) Protocol HIT-2000.^19^^,^^20^ The clinical and pathological information, including basic patient details, age, sex, metastatic status, Chang staging, postoperative residual, radiotherapy, chemotherapy, clinical risk and histopathological subtypes, was collected from medical records. The OS and PFS follow-up data were obtained from the patients’ electronic medical records. Ethical approval (KY2014-021-02) and patient consent for sample collection in this cohort were obtained from the Beijing Tiantan Hospital prior to craniotomy for all participants. For minor patients, informed consent is provided by their legal guardian (parent or other guardian) on their behalf.

### Molecular analysis

Molecular analysis was conducted retrospectively at a central laboratory. DNA was extracted from fresh-frozen and formalin-fixed, paraffin-embedded tissue samples. Samples were analysed using arrays (Illumina Infinium Methylation EPIC v2.0 Bead Chip) in accordance with the manufacturer’s instructions.^21^^,^^22^

### Procedures

Tissue microarrays were constructed from 1.5 mm punched cores from formalin-fixed paraffin-embedded blocks from tumour samples from 249 participants from the hospital, under guidance of the study pathologists, and were stored at room temperature protected from air. mIF staining for analyte markers (Supplementary Table S1) was done on tissue microarray sections using AlphaTSA Multiplex IHC kit (AlphaX Biotech, China). For assessing potential associations between immune marker with OS and PFS, the optimal threshold of density was calculated and used as the cut-off to stratify each MB case into high and low subgroups for analysis.^23^ Spatial analysis was performed considering the 2D XY coordinates of each marker. Tumour cells present within a maximum 30 μm radius from a given cell (of distinct phenotypes) were considered as “interacting”, as this radius represents an enhanced probability for cell–cell contact.^24^, ^25^, ^26^, ^27^

### Antibody and primary cell line validation

All antibodies and cell lines used in this study were validated prior to use. Each antibody was cited with its corresponding RRID to ensure traceability (Supplementary Table S1). The DAOY cell line was obtained from ATCC. All primary human MB cell lines established in our laboratory were verified by short tandem repeat (STR) profiling to confirm their human origin and identity and were routinely tested to be free of mycoplasma contamination. A detailed description of the experimental procedures is available in the Supplementary Methods.

### Outcomes

The primary endpoint was the association between the immune markers and MB outcomes. PFS was defined as the time between initial diagnosis of MB and progression of tumour occurrence (any aspect) or death (from any cause). OS was defined as the time from initial diagnosis of MB to death (from any cause). Both endpoints were assessed for the whole cohort. The exploratory analysis included searching for informative immune markers for the PFS and OS of patients with MB, as well as analysing the effects of different molecular groups, histopathological subtypes, and clinical characteristics on immune cell infiltration (Supplementary Table S2).

### Statistical analysis

Categorical features were analysed using chi-square and Fisher’s exact tests (for expected counts <5). For continuous data, ANOVA or the Kruskal–Wallis rank-sum test was used to compare means and medians, respectively. The significance threshold for all statistical tests in this study was set at *p* < 0.0500. Differences among multiple molecular subgroups were analysed using one-way ANOVA, and post-hoc pairwise comparisons were adjusted for multiple testing using the Bonferroni correction. Survival analyses were conducted using Kaplan–Meier curves and Log-rank tests, with a 95% CI reported. The survminer package was used to categorise the data into high- and low-density groups based on the optimal threshold obtained from surv_cutpoint for survival analysis. Additionally, univariate Cox regression models were executed using the coxph function from the survival package. A multivariable Cox proportional hazards regression model was employed to evaluate the association between immune markers and overall survival. Each immune marker, treated as a binary variable, was incorporated into the model alongside clinical covariates, including age at diagnosis, gender, molecular subtype, metastatic status, and histological type. The findings are presented as hazard ratios (HR), 95% confidence intervals (CI), and *p*-values. The proportional hazards assumption was assessed using Schoenfeld residuals, utilising the cox.zph function from the survival package. Analyses were performed using R version 4.2.1 (2022-06-23). The following R packages (with versions) were used: survminer version 0.5.0, survival version 3.8.3. Analyses and visualizations were performed using GraphPad Prism 8 and FlowJo.

### Role of funders

The funders were not involved in the study’s design, implementation, analysis, or interpretation, nor did they participate in writing the manuscript or deciding to submit it for publication.

## Results

### Patient and tumour characteristics

Between 2014 and 2020, 249 surgically resected MB samples were collected from paediatric patients, including 167 male (67.068%) and 82 female patients (32.932%), as shown in Table 1 and Supplementary Table S3. Regarding risk stratification of MB based on clinical criteria, 166 were average-risk (66.667%) and 83 were high-risk (33.333%) (Supplementary Table S4). The median age was 7 years (Q1 was 5 and Q3 was 10). By the cut-off date in February 2025, 207 were alive (83.133%). The overall survival rate for the entire cohort was 83.133%, while the progression-free survival rate was 72.290% at the last follow-up. Patients were initially categorised into one of four previously reported molecular groups, including the G3 molecular group (42 patients; 16.867%; median age 5 years), G4 molecular group (120; 48.193%; 7 years), WNT molecular group (32; 12.851%; 8 years), and the SHH molecular group (55; 22.088%; 7 years). The patients were additionally diagnosed based on traditional pathology into one of four histopathological subtypes, including classic (169 patients; 67.871%; median age 7 years), DN (57 patients; 22.892%; median age 8 years), LC/A (10 patients; 4.016%; median age 7 years), and MBEN (13 patients; 5.221%; median age 7 years).

**Table 1:** Molecular groups of the 249 patients with paediatric medulloblastoma whose tumour specimens were included in our study.

Characteristic	All patients	Medulloblastoma				*p* value
		G3	G4	WNT	SHH	
Frequency N (%)	249	42 (16.867%)	120 (48.193%)	32 (2.851%)	55 (22.088%)	
Age						
Mean (SD)	7.448 (3.394)	5.667 (2.729)	7.971 (3.343)	8.75 (3.376)	7.029 (3.503)	<0.0001∗∗∗∗
Median (Q1, Q3)	7 (5, 10)	5 (4, 7.25)	8 (6, 10)	8.5 (6, 11.25)	7 (4.25, 9)	
Sex						
M	167 (67.068%)	31 (18.563%)	82 (49.102%)	12 (7.186%)	42 (25.150%)	0.0014∗∗
F	82 (32.932%)	11 (13.415%)	38 (46.341%)	20 (24.390%)	13 (15.854%)	
Total	249 (100.000%)	42 (16.867%)	120 (48.193%)	32 (12.851%)	55 (22.088%)	
Histological subtypes						
Classic	169 (67.871%)	35 (20.710%)	100 (59.172%)	28 (16.568%)	6 (3.550%)	<0.0001∗∗∗∗
DN	57 (22.892%)	3 (5.263%)	20 (35.088%)	1 (1.754%)	33 (57.895%)	
LC/A	10 (4.016%)	4 (40.000%)	0 (0.000%)	3 (30.000%)	3 (30.000%)	
MBEN	13 (5.221%)	0 (0.000%)	0 (0.000%)	0 (0.000%)	13 (100.000%)	
Chang staging						0.0025∗∗
Average	166 (66.667%)	19 (11.446%)	86 (51.807%)	34 (20.482%)	27 (16.265%)	
High	83 (33.333%)	23 (27.711%)	34 (40.964%)	5 (6.024%)	21 (25.301%)	
Metastatic status						
M0	170 (68.273%)	19 (11.176%)	89 (52.353%)	27 (15.882%)	35 (20.588%)	0.0080∗∗
M+	79 (31.727%)	23 (29.114%)	31 (39.241%)	5 (6.329%)	20 (25.316%)	
Total	249 (100.000%)	42 (16.867%)	120 (48.193%)	32 (12.851%)	55 (22.088%)	
Postoperative residual						1.0000
<1.5 cm^2^	249 (100.000%)	42 (16.867%)	120 (48.193%)	32 (12.851%)	55 (22.088%)	
≥1.5 cm^2^	0 (0.000%)	0 (0.000%)	0 (0.000%)	0 (0.000%)	0 (0.000%)	
Total	249 (100.000%)	42 (16.867%)	120 (48.193%)	32 (12.851%)	55 (22.088%)	
Radiotherapy						
Y	248 (99.598%)	42 (16.935%)	120 (48.387%)	32 (12.903%)	55 (22.177%)	0.1573
N	1 (0.402%)	1 (100.000%)	0 (0.000%)	0 (0.000%)	0 (0.000%)	
Total	249 (100.000%)	42 (16.867%)	120 (48.193%)	32 (12.851%)	55 (22.088%)	
Chemotherapy						0.1573
Y	248 (99.598%)	42 (16.935%)	120 (48.387%)	32 (12.903%)	55 (22.177%)	
N	1 (0.402%)	1 (100.000%)	0 (0.000%)	0 (0.000%)	0 (0.000%)	
Total	249 (100.000%)	42 (16.867%)	120 (48.193%)	32 (12.851%)	55 (22.088%)	
Survival status						
Alive	207 (83.133%)	29 (14.010%)	102 (49.275%)	32 (15.459%)	44 (21.256%)	0.0034∗∗
Decease	42 (16.867%)	13 (30.952%)	18 (42.857%)	0 (0.000%)	11 (26.190%)	

The statistical analysis involved chi-square tests to determine *p* values for categorical variables. Fisher's exact test was employed specifically for categorical variables where expected counts were below five. For continuous data, comparisons of mean values were conducted using ANOVA tests (∗∗*p* < 0.0100, ∗∗∗∗*p* < 0.0001).

### Outcomes by molecular groups

The median follow-up was 5.68 years from MB diagnosis. Log-rank tests revealed highly significant differences in molecular groups related to OS (*p* = 0.0041, Log-rank test) and PFS (*p* < 0.0001, Log-rank test). Favourable survival outcomes for the WNT group were confirmed in a Kaplan–Meier analysis (Supplementary Fig. S1A and S1B). The estimated 5-year overall survival rate was 69.048% for G3, 85.000% for G4, 80.000% for SHH, and 100.000% for WNT. No associations were detected between survival outcome with age and gender. Log-rank tests revealed no significant differences in histopathological subtypes related to OS (*p* = 0.1600, Log-rank test) and PFS (*p* = 0.1660, Log-rank test) (Supplementary Fig. S1C and S1D). The estimated 5-year overall survival rate was 81.657% for classic, 70.000% for LC/A, 76.923% for MBEN, and 91.228% for DN.

### Multiplex immunofluorescence panels for characterising immune cell populations in MB

**Fig. 2:**
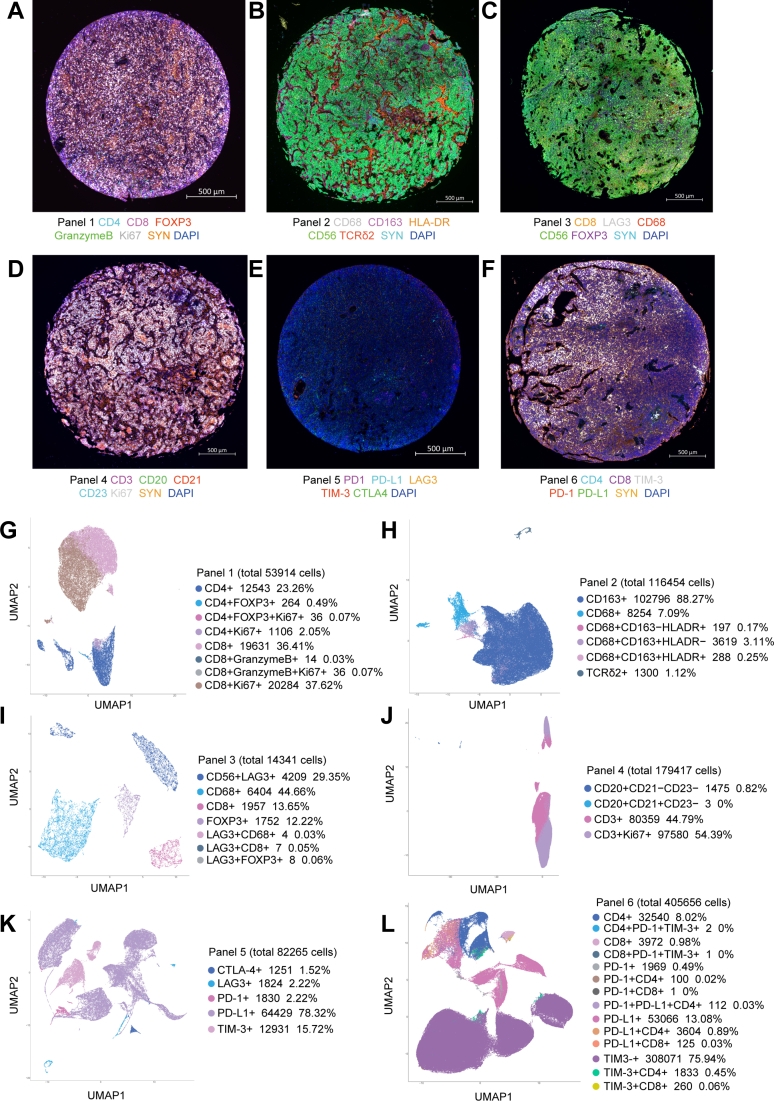
Representative examples for mIF analysis of MB tissue microarray specimens. The images are organised as follows: (A) Panel 1: CD4, CD8, PD-1, FOXP3, Granzyme B, Ki67, SYN, and DAPI. (B)Panel 2: CD68, CD163, HLA–DR, CD56, TCRδ2, SYN, and DAPI. (C) Panel 3: CD8, LAG-3, CD68, CD56, Foxp3, SYN, and DAPI. (D) Panel 4: CD3, CD20, CD21, CD23, Ki67, SYN, and DAPI. (E) Panel 5: PD-1, PD-L1, LAG-3, TIM-3, CTLA-4, and DAPI. (F) Panel 6: CD4, CD8, PD-1, TIM-3, PD-L1, SYN, and DAPI. Scale bars: A–F, 500 μm. (G–L) UMAP of cell types identified using mIF Panels 1–6 (n = 249). Each UMAP (G–L) was generated by pooling all immune cells from all patients within the corresponding staining panel.

Given the relative paucity of clinical data for adaptive immune cells in MB, we designed a series of six multiplex IF panels to profile T cell subsets, Macrophages, NK cells, TLSs, and immune checkpoints across all 249 samples (Fig. 2A–F and Supplementary Fig. S2A). Briefly, for all panels, synaptophysin positive (SYN+) status was considered indicative of tumour cells,^4^ and the SYN signal was used to delineate the “tumour region” in each sample; SYN− status was indicative of stromal cells and was used to delineate “adjacent normal regions”. Panel 1 was designed to identify T lymphocytes (CD4+ and CD8+), activated cytotoxic T lymphocytes (CD8+Granzyme B+), T-regulatory cells (CD4+FOXP3+), and the proliferation marker Ki67. Panel 2 assesses infiltration by γδT cells (TCRδ2+), NK cells (CD56+SYN−), type I macrophages (CD68+CD163-HLADR+), type II macrophages (CD68+CD163+HLADR−), and Mmix cells (CD68+CD163+HLADR+). Panel 3 complements Panels 1 and 2, as it was designed to assess LAG-3 expression on cytotoxic T cells (CD8+LAG-3+), macrophages (CD68+LAG-3+), NK cells (CD56+LAG-3+), Treg cells (FOXP3+LAG-3+) and tumour cells (SYN+LAG-3+). Panel 4 assesses the presence of early tertiary lymphoid structures (CD20+CD21−CD23− cells), primary tertiary lymphoid structures (CD20+CD21+CD23− cells), and secondary tertiary lymphoid structures (CD20+CD21+CD23+ cells). Panel 5 evaluates expression of immune checkpoints including PD1, PD-L1, CTLA4, TIM-3, and LAG-3. Panel 6 assesses PD1, PD-L1, and TIM-3 specifically as expressed on immune cells (CD4+PD-1+, CD4+TIM-3+, CD8+PD-1+ and CD8+TIM-3+) and tumour cells (SYN+PD-1+, SYN+TIM-3+ and SYN+PD-L1+).

Due to the relatively low density of immune cells in the brain, we aggregated the immune cells and conducted dimensionality reduction for each panel independently. We tallied the total numbers for immune cell types in terms of the four molecular groups. Dimension reduction plots were used to help visualise and identify the different cell phenotypes detected in each multiplex IF panel based on marker co-expression (Fig. 2G–L).

### Characterisation of CD3, CD4, macrophages, and NK cells in the context of MB

**Fig. 3:**
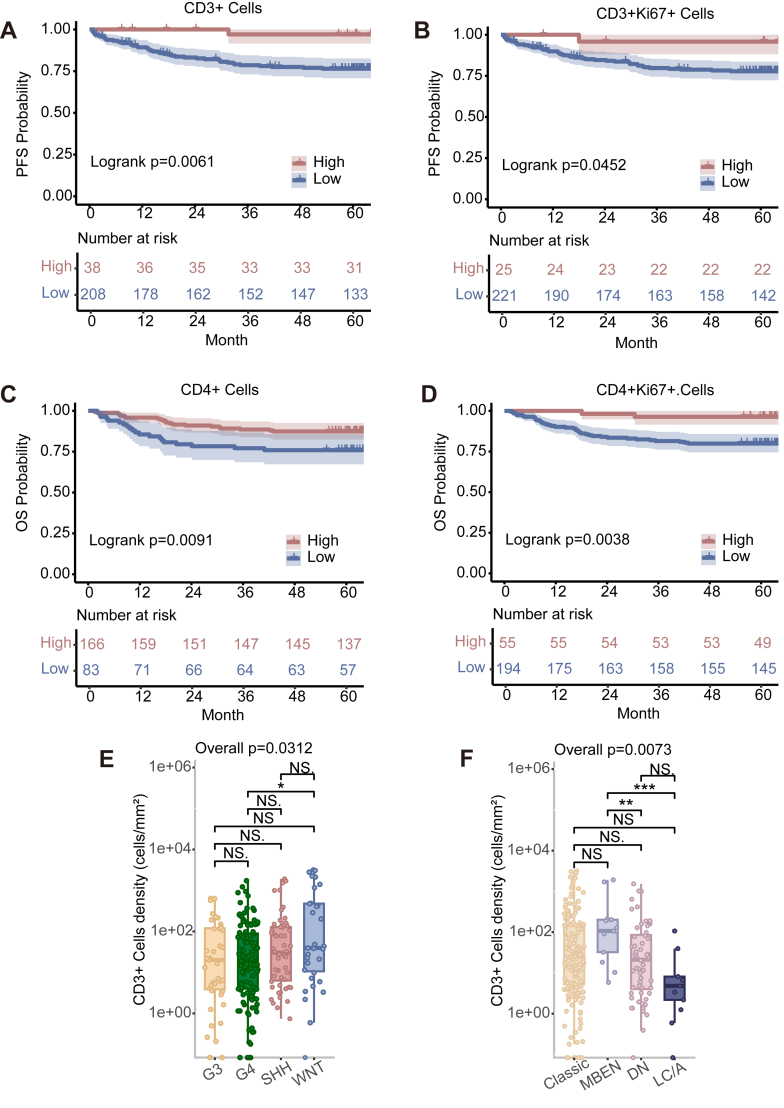
Survival analysis by T cell. (A–D) Kaplan–Meier curves for OS or PFS in terms of T cells. Kaplan–Meier curves for PFS for the overall study cohort in terms of (A) CD3+ T cells, (B) CD3+Ki67+ cells. Kaplan–Meier curves for OS in terms of (C) CD4+ T cells and (D) CD4+Ki67+ cells. The optimal threshold value of each variable was used as the surv_cutoff to define high and low subgroups for analysis. Log-rank *p*-values are reported in each graph. (E, F) Variation in the numbers of CD3+ cells among (E) molecular groups and (F) histopathological subtypes. Number of patients: Group 3 (42); Group 4 (120); WNT (32); SHH (55); Classic (169); DN (57); LC/A (10); MBEN (13). Data are presented as cell density (number of cells/mm^2^). Differences among multiple groups were analysed using one-way ANOVA followed by post-hoc multiple comparisons, with *p*-values adjusted using the Bonferroni correction (∗*p* < 0.05, ∗∗*p* < 0.01, ∗∗∗*p* < 0.001, ∗∗∗∗*p* < 0.0001).

We initially used the mIF data to examine potential differences in the distribution of particular immune cell types for the overall study cohort and among the molecular groups, starting with T cells. We analysed the PFS and OS of all panels, identifying those with statistically significant differences. In the overall study cohort, a Log-rank test analysis indicated that a higher density of CD3+ T cells (HR^low vs high^ 5.15 [95% CI 1.250–21.184], *p* = 0.0233, Wald test) in tumours was associated with a longer PFS (Fig. 3A); a similar trend was detected CD3+Ki67+ (HR^low vs high^ 3.29 [95% CI 0.800–13.554], *p* = 0.0989, Wald test) (Fig. 3B). A similar pattern was observed for CD4+ T cells (HR^low vs high^ 2.19 [95% CI 1.197–4.016], *p* = 0.0110, Wald test) (Fig. 3C), CD4+Ki67+ T cells (HR^low vs high^ 6.25 [95% CI 1.509–25.843], *p* = 0.0115, Wald test) (Fig. 3D) in OS. Beyond assessing potential associations with prognosis, our subsequent pairwise comparisons showed significantly more CD3+ T cells in the WNT group tumours than in the G4 group (*p* < 0.0500, ANOVA) (Fig. 3E). This may imply that the infiltration capacity of CD3+ T cells is poor in G3 group with relatively poor clinical prognosis, and the reduced proliferative capacity of CD3+ cells also limited their anti-tumour function. Comparison of histopathological subtypes revealed a conspicuously low number of CD3+ T cells in the LC/A subtype (*p* < 0.0100, ANOVA) (Fig. 3F, Supplementary Tables S5–S7).

**Fig. 4:**
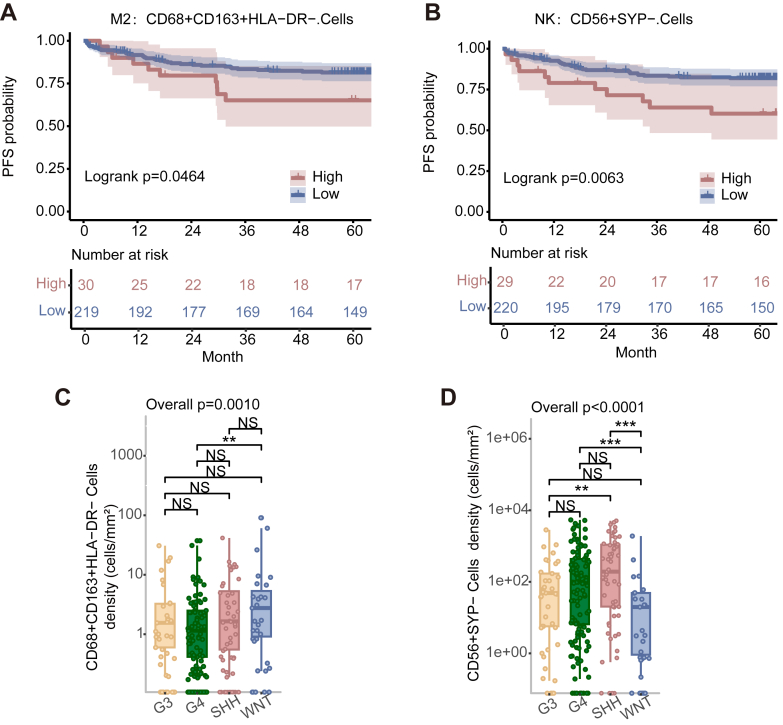
Survival analysis by macrophage cells. (A, B) Kaplan–Meier curves for PFS for the overall study cohort in terms of (A) type II macrophages, (B) NK cell, the optimal threshold value of each variable was used as the surv_cutoff to define high and low subgroups for analysis. Log-rank *p*-values are reported in each graph. (C, D) Variation in the numbers of (C) type II macrophages and (D) NK cell among molecular groups. Number of patients: Group 3 (42); Group 4 (120); WNT (32); SHH (55); Classic (169); DN (57); LC/A (10); MBEN (13). Data are presented as cell density (number of cells/mm^2^). Differences among multiple groups were analysed using one-way ANOVA followed by post-hoc multiple comparisons, with *p*-values adjusted using the Bonferroni correction (∗*p* < 0.05, ∗∗*p* < 0.01, ∗∗∗*p* < 0.001, ∗∗∗∗*p* < 0.0001).

The Log-rank tests indicated that a higher density of type II macrophages in tumours was associated with a worse PFS (HR^low vs high^ 0.50 [95% CI 0.250–1.003], *p* = 0.0509, Wald test) (Fig. 4A). The Log-rank tests also indicated that a higher density of NK cells in tumours was associated with a worse PFS (HR^low vs high^ 0.40 [95% CI 0.207–0.792], *p* = 0.0083, Wald test) (Fig. 4B). A subsequent pairwise comparison among molecular groups showed significantly fewer type II macrophages in the G4 group tumours than in WNT (*p* < 0.0010, ANOVA) groups (Fig. 4C). These trends may indicate that the infiltration ability of type II macrophages in WNT is relatively stronger than in the other groups, so immunotherapy targeting macrophages may have more potential therapeutic potential for the WNT population. The comparison among molecular groups revealed a conspicuously high number of NK cells in the SHH group (*p* < 0.0001, ANOVA) (Fig. 4D and Supplementary Tables S5–S7).

### The presence of tertiary lymphoid structures in MB

**Fig. 5:**
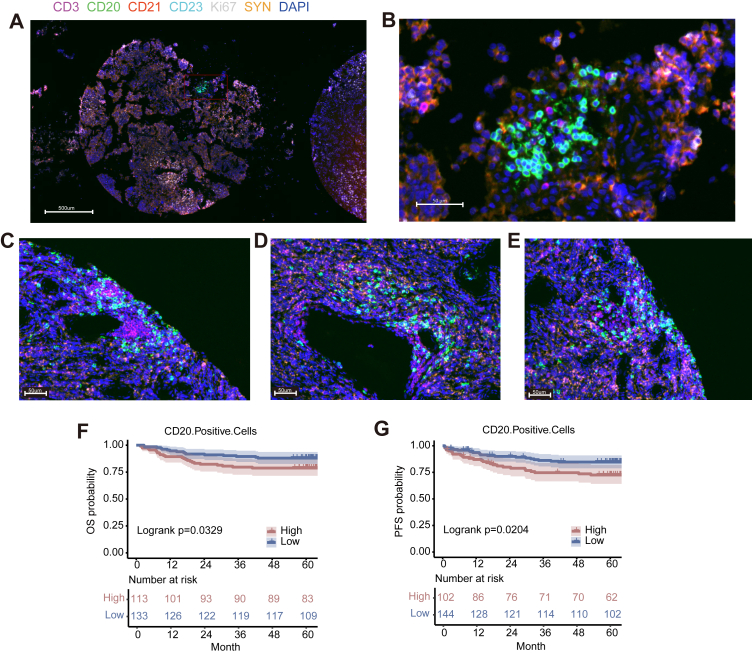
Survival analysis by TLSs. (A, B) Immunofluorescence image of early TLSs (CD20+CD21−CD23−) in one patient was of the classic subtype and WNT group. (C–E) Immunofluorescence image of early TLSs (CD20+CD21−CD23−) in another patient was of the DN subtype and G4 group. Scale bars: A = 500 μm; B–E = 50 μm. (F, G) Kaplan–Meier curves for (F) OS or (G) PFS in terms of CD20+ B cells. The optimal threshold value of each variable was used as the surv_cutoff to identify high and low subgroups for analysis. Log-rank tests were performed to determine significance; p-values are reported in each graph. Data are presented as cell density (number of cells/mm^2^). Differences among multiple groups were analysed using one-way ANOVA followed by post-hoc multiple comparisons, with *p*-values adjusted using the Bonferroni correction (∗*p* < 0.05, ∗∗*p* < 0.01, ∗∗∗*p* < 0.001, ∗∗∗∗*p* < 0.0001).

TLSs are organised, ectopic lymphoid structures formed by the accumulation of lymphocytes in non-lymphoid organs under pathophysiological circumstances.^23^ TLSs are understood as sites for interactions between tumour cells and immune cells,^28^ and their presence can be informative regarding patient prognosis.^29^ TLSs were identified as aggregates of lymphocytes having histological features with analogous structures to that of lymphoid tissue with germinal centres including CD20+ B cells, CD3+ T cells, CD21+ follicular dendritic cells and CD23+ germinal centre (GCs) appearing in the tumour area.^28^ We defined TLS-positive tumours as those containing CD3+ T-cell aggregates colocalised with CD20+ B cells, covering an area >7000 μm^2^ (as measured by Halo10 software), consistent with prior reports.^30^ Briefly, our mIF Panel 4 uses CD3, CD20, CD21, and CD23 as markers to assess the presence and maturity of TLSs. We defined TLSs enriched with CD20+ B cells and CD3+ T lymphocytes as early-stage TLSs. Primary follicle-like TLSs were characterised as CD20+ B cells, CD3+ T cell clusters, and CD21+ DCs with CD23− GCs; secondary follicle-like TLSs were characterised as CD20+ B cells, CD3+ T cell clusters, CD21+ DCs, and CD23+ GCs. Notably, Panel 4 revealed the presence of intact early TLSs in 2 of the 249 examined patients with MB, and two patients exhibited distinct CD20+ clusters, surrounded by CD3+ cells, with CD21− and CD23−. Patient one, a female aged 11, was of the classic histopathological subtype and the WNT molecular group. She presented with one TLS cluster comprising more than 50 aggregated CD20+ B cells. Patient two, a female aged 12 (DN subtype, G4 group) had three TLS clusters, each containing more than 50 CD20+ B cells (Fig. 5A–E). We found that higher CD20+ B cells were associated with a worse PFS (HR^low vs high^ 0.51 [95% CI 0.291–0.902], *p* = 0.0205, Wald test) and OS (HR^low vs high^ 0.50 [95% CI 0.271–0.941], *p* = 0.0314, Wald test) (Fig. 5F and G).

### Assessing spatial distances between various cellular populations in MB

**Fig. 6:**
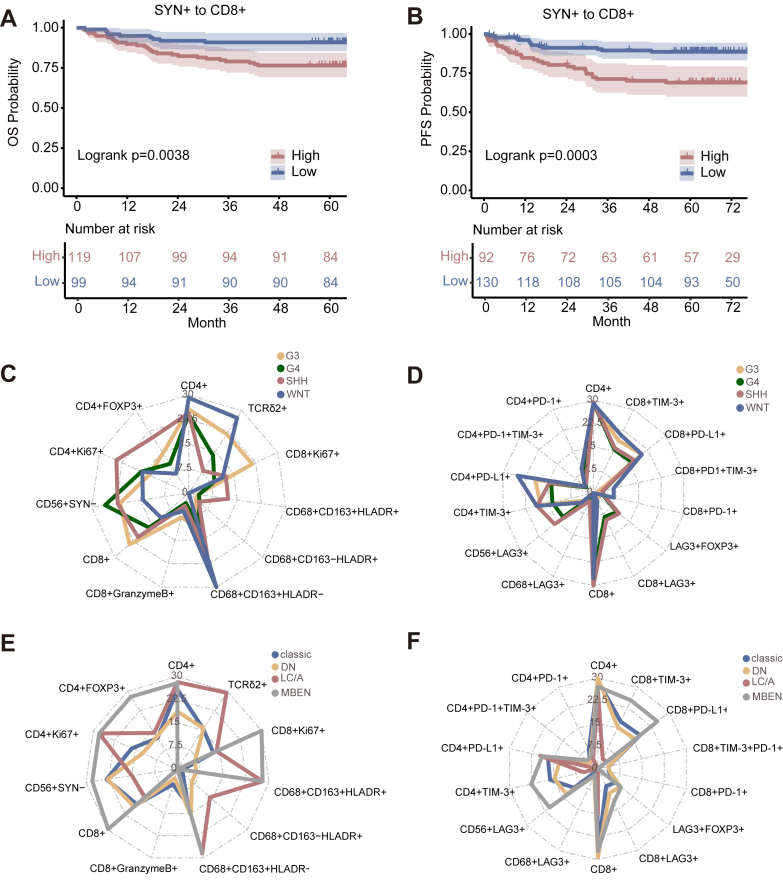
Spatial analysis. (A) Kaplan–Meier curves for OS according to the spatial distance between SYN+ cells and CD8+ cells. (B) Kaplan–Meier curves for PFS according to the spatial distance between SYN+ cells and CD8+ cells. The optimal threshold value of each variable was used as the cut-off to define high and low subgroups for analysis. Log-rank p-values are reported in each graph. (C, D) Radar chart depicting the distance from SYN+ cells to immune cells among the molecular groups. (E, F) Radar chart depicting the distance from SYN+ cells to immune cells among the histopathological subtypes.

Investigating interactions between malignant cells and tumour-associated immune cells through spatial distribution can help identify potential trends that influence tumour progression, relapse, and outcomes.^31^ We found that several spatial interactions in Panels 1, Panel 2, Panel 3, and Panel 6 (as evaluated by distance analysis; see Methods) were associated with patient outcomes. Greater distance between SYN+ and CD8+ T cells was associated with worse OS (HR^low vs high^ 0.45 [95% CI 0.21–0.956], *p* = 0.0378, Wald test, Fig. 6A), and PFS (HR^low vs high^ 0.33 [95% CI 0.172–0.621], *p* = 0.0006, Wald test, Fig. 6B).

We analysed the distribution from SYN+ tumour cells to immune cells within 30 μm distance across different molecular groups (Fig. 6C and D) and histopathological subtypes (Fig. 6E and F). And there were significant differences for the cell numbers of SYN+ to CD68+LAG-3+ cells (*p* < 0.0001, ANOVA), SYN+ to FOXP3+LAG-3+ cells (*p* = 0.0008, ANOVA), and SYN+ to CD8+PD-1 cells (*p* < 0.0001, ANOVA) between four molecular groups (Supplementary Tables S8–S9). And there were significant differences among the histopathological subtypes for the cell numbers of SYN+ to CD68+CD163+ HLADR+ macrophage cells (*p* = 0.0006, ANOVA), SYN+ to CD56+LAG-3+ cells (*p* = 0.0020, ANOVA), and SYN+ to CD8+TIM-3+ T cells (*p* = 0.0290, ANOVA) (Supplementary Tables S8–S9).

### Characterising of immune checkpoint expression in MB

**Fig. 7:**
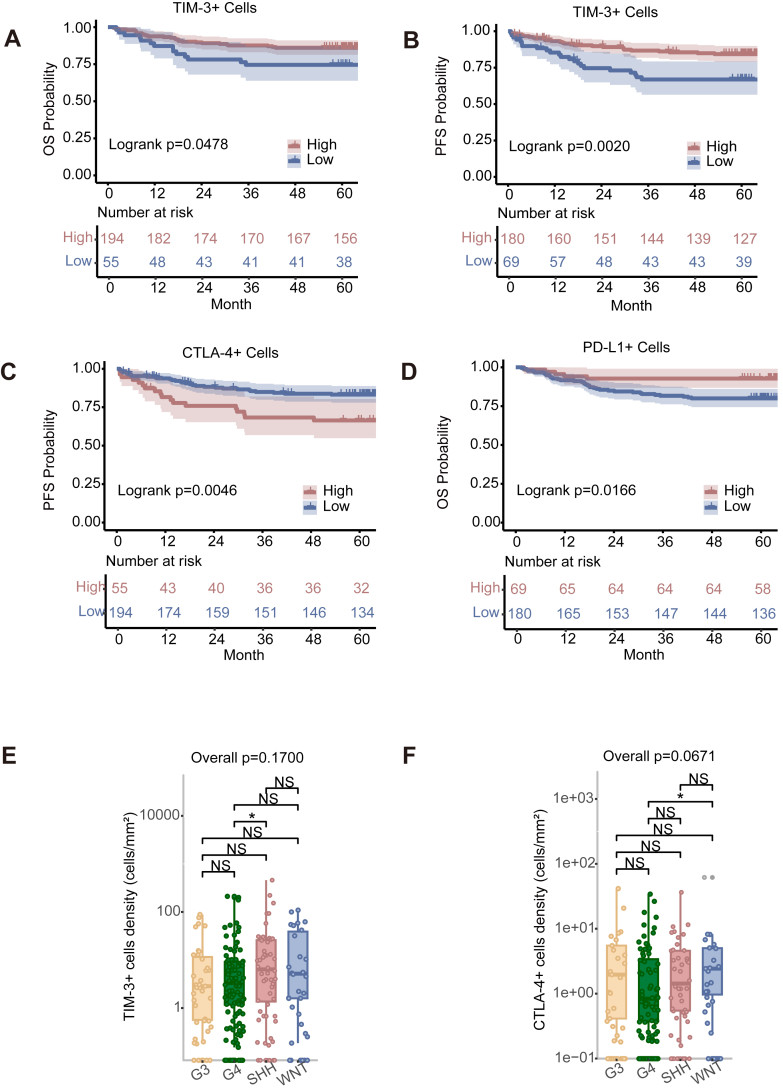
Survival analysis by immune checkpoint cells. (A–D) Kaplan–Meier curves for OS in terms of (A) TIM-3+ cells and (D) PD-L1+ cells. Kaplan–Meier curves for PFS in terms of (B) TIM-3+ cells and (C) CTLA-4+ cells. The optimal threshold value of each variable was used as the surv_cutoff to identify high and low subgroups for analysis. Log-rank tests were performed to determine significance; *p*-values are reported in each graph. (E, F) Variation in the numbers of TIM-3+ cells, and CTLA-4+ cells among the molecular groups. Number of patients: Group 3 (42); Group 4 (120); WNT (32); SHH (55); Classic (169); DN (57); LC/A (10); MBEN (13). Data are presented as cell density (number of cells/mm^2^). Differences among multiple groups were analysed using one-way ANOVA followed by post-hoc multiple comparisons, with *p*-values adjusted using the Bonferroni correction (∗*p* < 0.05, ∗∗*p* < 0.01, ∗∗∗*p* < 0.001, ∗∗∗∗*p* < 0.0001).

We assessed potential associations between immune checkpoint molecules and prognosis. An increased number of TIM-3+ cells (HR^low vs high^ 1.89 [95% CI 0.996–3.593], *p* = 0.0516, Wald test) (Fig. 7A) and PD-L1 cells (HR^low vs high^ 2.97 [95% CI 1.166–7.549], *p* = 0.0225, Wald test) (Fig. 7D) were associated with improved OS. An increased number of TIM-3+ cells (HR^low vs high^ 2.37 [95% CI 1.347–4.155], *p* = 0.0027, Wald test) (Fig. 7B) were associated with improved PFS, while a decline in PFS was observed in CTLA-4+ cells (HR^low vs high^ 0.44 [95% CI 0.247–0.790], *p* = 0.0059, Wald test) (Fig. 7C).

For molecular groups, pairwise comparisons revealed significantly more TIM-3+ cells in the SHH group than in the G4 group (Fig. 7E), as well as significantly more CTLA-4+ cells in the WNT group than in the G4 group (Fig. 7F and Supplementary Tables S5–S7).

### Evaluating tumour cell checkpoint expression and associations with MB prognosis

We identified that an increased number of SYN+TIM-3+ tumour cells was associated with a longer OS (HR^low vs high^ 3.57 [95% CI 1.104–11.561], *p* = 0.0336, Wald test) (Supplementary Fig. S3A), and PFS (HR^low vs high^ 2.24 [95% CI 1.242–4.034], *p* = 0.0073, Wald test) (Supplementary Fig. S3B). A similar trend was observed for SYN+PD-L1+ tumour cells (HR^low vs high^ 2.49 [95% CI 1.356–4.573], *p* = 0.0033, Wald test) (Supplementary Fig. S3C). Pairwise comparisons of molecular groups showed more SYN+TIM-3+ tumour cells (Supplementary Fig. S3D) and SYN+PD-L1+ tumour cells (Supplementary Fig. S3F) in the WNT group than other groups. In histopathological subtypes, significantly more SYN+TIM-3+ tumour cells (Supplementary Fig. S3E) and SYN+PD-L1+ tumour cells (Supplementary Fig. S3G) were observed in the MBEN subtype than other subtypes. Specifically, we evaluated TIM-3 expression in human primary MB using flow cytometry and immunoblotting and noted strong TIM-3 expression on MB tumour cells (Supplementary Fig. S3H).

### Multivariate analysis

Multivariate analysis was conducted using Cox proportional hazards regression models. Then a multivariate regression analysis indicated that CD20+ B cells were informative as independent prognostic factors for OS (HR^high vs low^ 2.30 [95% CI 1.20–4.39], *p* = 0.0120, Wald test) and PFS (HR^high vs low^ 2.38 [95% CI 1.32–4.32], *p* = 0.0041, Wald test) (Supplementary Table S10). Additionally, a multivariate regression analysis indicated that CTLA-4+ cells were informative as independent prognostic factors for PFS (HR^high vs low^ 2.10 [95% CI 1.11–3.95], *p* = 0.0218, Wald test) (Supplementary Table S11).

### External data validation

To assess the prognostic value of immune markers, we employed a discovery-validation approach, using mIF data serving as the discovery cohort and external RNA-seq data as the validation cohort.^17^ The external validation was performed using a well-characterised multicentre MB RNA-seq dataset curated by Taylor et al.^32^ The cohort contains 806 patients with available clinical, pathological, and survival data. Only patients with complete molecular subgrouping and survival data were included in this analysis. The original data were accessed from GSE85218. Briefly, we selected 409 patients (aged up to 18 years at diagnosis) who had available clinical information, including molecular groups, histopathological subtypes, and M-stage metastasis. After excluding those in the NOS (not otherwise specified) and WNT groups (at the time of the statistical analysis cut-off, patients in the WNT subgroup of MB were still alive), 403 patients remained. Among them, 381 patients had complete OS information, which were used for multivariate Cox model validation. Additionally, 262 patients had complete PFS information, which were used for multivariate Cox model validation. We aimed to validate our findings in the eligible cases (381 OS patients; 262 PFS patients) in the external cohort. Through association analysis, the prognostic value of B cells (CD20) was confirmed in cases from the external dataset, where B cell density was treated as a categorical variable (high versus low). The results showed a hazard ratio of 2.21 (95% CI 1.341–3.651, *p* = 0.0020, Wald test) in OS and HR of 2.15 (95% CI 1.28–3.61, *p* = 0.0038, Wald test) in PFS. Similar to the discovery cohort, high expression levels of CTLA4 in the external dataset are associated with a poor PFS prognosis, exhibiting a HR of 1.83 (95% CI 1.100–3.020, *p* = 0.0189, Wald test) (Supplementary Tables S12–S15).

## Discussion

mIF has demonstrated significant potential for the discovery and validation of immune biomarkers due to its capacity to simultaneously visualise multiple immune cell markers while preserving the spatial structure of tissues.^27^^,^^33^^,^^34^ Currently, flow cytometry and RNA sequencing are employed to detect immune cell infiltration in MB. The application of mIF technology allows us to highlight the prognostic significance of spatial interactions among immune biomarkers within the context of MB—interactions that cannot be adequately assessed through immunohistochemistry (IHC) or bulk gene expression techniques. Indeed, given the critical role of the topological distribution of immune cells within the tumour microenvironment, merely analysing the density of immune cells or their activation status is insufficient for a comprehensive characterisation of the immune microenvironment in MB.

The immune microenvironment of central nervous system tumours is constrained by the presence of the blood–brain barrier, which limits the infiltration of immune cells and the delivery of therapeutic agents. Recent studies have discovered the existence of functional lymphatic vessels within the dural sinuses under physiological conditions.^35^ There is a gradual understanding emerging about the immune microenvironment in MB that has enhanced the feasibility of undertaking immunotherapeutic interventions.^36^ The present study provides an empirical assessment of infiltrating immune cells in a large cohort of MB cases and supported exploration of their associations with patient outcomes. Further, the use of mIF technology allowed us to evaluate how interactions among immune and cancer cells affect MB prognosis.

In this study, we performed an in-depth analysis of the immune markers in 249 cases of MB, offering valuable insights into the characteristics of immune cell infiltration and its potential impact on the prognosis of MB. This work extends and validates prior, smaller studies, confirming the broader influence of immune cell populations on MB outcomes.^36^, ^37^, ^38^, ^39^ Additionally, we observed significant differences in immune cell infiltration patterns across various molecular groups and histopathological subtypes, underscoring the complex role of the immune microenvironment in MB.

In our study, higher densities of intratumoural CD3+ T cells, CD3+Ki67+ T cells, CD4+ T cells, CD4+Ki67+ T cells were associated with prolonged OS, a strong association consistent with established evidence of T cells in anti-tumour immunity.^40^^,^^41^ When considering the role of macrophages in MBs, the M1/M2 dichotomization represents an oversimplification, and a spectrum of activation states exists with many cells displaying a mixed phenotype (Mmix) and M1-and M2-polarized macrophages representing the extremes of this spectrum.^36^ We found that patients with a high M2 cell count had a poor short-term prognosis could be because M2 macrophages mainly secrete anti-inflammatory cytokines such as Arginase-I, IL-10, and TGF-β, which help reduce inflammation, promote tumour growth, and support immunosuppression, consistent with the well-established pro-tumour effect of M2 macrophages in various tumours.^42^^,^^43^

Studies have shown that the immune mesenchymal-enriched (IME) subtype in IDH-mutant gliomas exhibits higher levels of immune cell infiltration, yet patients experience poor survival outcomes.^44^ A comparable phenomenon was observed in our MB study: although immune fluorescence detection revealed a high density of NK cells, these patients exhibited poor prognoses. This discrepancy may arise from the possibility that, despite the elevated number of NK cells, their functionality could be impaired by the tumour microenvironment (inhibitory molecules, checkpoint inhibition, immune cell exhaustion, etc.), or from the presence of immune-suppressive factors in the tumour vicinity (regulatory T cells, myeloid-derived suppressor cells, and high expression of inhibitory cytokines), which may prevent NK cells from effectively exerting their anti-tumour effects.^45^, ^46^, ^47^ Consequently, a high NK cell density in MB may not signify a favourable immune response but rather indicate a suppressed or dysregulated immune state. This finding also implies that NK cell density alone may not suffice to predict the prognosis of patients with MB. Future research should further investigate the activation status of these NK cells, along with inhibitory/exhaustion markers, and the subtypes, functions, and conditions of the tumour microenvironment to more accurately elucidate the relationship between immune infiltration and patient outcomes.

Additionally, we identified early TLSs in MB, which had not been previously reported. The formation of TLSs within the tumour microenvironment, rather than in traditional secondary lymphoid organs, is a relatively new area of interest in cancer immunology.^28^ These findings suggest that TLSs may play an important role in initiating immune responses within the tumour, a process traditionally thought to occur only in lymph nodes.^29^^,^^31^ Consistent with previous studies linking B cells to unfavourable clinical outcomes in cancers such as renal carcinoma, prostate cancer, and glioblastoma,^48^^,^^49^ our study found that a higher density of CD20+ B cells was associated with poorer survival outcomes. Research suggests that B cells may play a dual role in certain tumours.^50^ The relationship between the number of B cells within a tumour and prognosis remains controversial. Some studies indicate that an increase in B cells may be associated with a favourable prognosis, while others suggest that B cells may promote tumour progression.^48^^,^^51^^,^^52^ Therefore, infiltrating B cells and TLSs may represent distinct types of immune responses, warranting further investigation.

TIM-3 and CTLA-4 were also considered by us as prognostic indicators for survival. However, Tim-3 may also be a valuable prognostic indicator in glioma research.^53^ A previous study reported that DIPG tumour cells express TIM-3, indicating its potential therapeutic target for DIPG. Studies using methylation data and immune deconvolution algorithms have inferred elevated TIM-3 expression. These methods estimate immune composition but do not clearly define the spatial localization or cellular origin of TIM-3.^39^ One study observed an increase in TIM-3 in IBA-1^+^ tumour-associated macrophages using human transcriptomic data and multiplex immunofluorescence. However, it did not include SYN as a marker for MB tumour cells, thus failing to visually confirm the spatial expression pattern of TIM-3 in tumour cells.^54^ In contrast, our study directly observed the co-localisation of TIM-3 and SYN through multiplex immunofluorescence, labelled with SYN, demonstrating that TIM-3 is expressed not only in infiltrating immune cells but also in MB tumour cells themselves. In a previous small cohort, the investigators found that CTLA-4 expression was absent in MB tumours in the cohort, possibly because the absence may reflect the limited sample size.^55^ Current clinical trials targeting PD-1 and CTLA-4 have demonstrated that monotherapy with PD-1 inhibition does not improve prognosis. In contrast, the combination blockade of PD-1 and CTLA-4 shows potential benefits in PFS.^56^^,^^57^ Notably, we observed that CTLA-4^+^ cell infiltration is significantly associated with poor PFS, indicating that CTLA-4-related immune suppression may be a dominant immune escape mechanism in central nervous system tumours. This finding may elucidate the reduced efficacy of PD-1 monotherapy and provides a biological rationale for investigating combination checkpoint inhibition strategies in paediatric brain tumours.

In spatial analysis, the proximity of CD8+ T cells to SYN+ tumour cells was significantly associated with survival. This finding indicates that the spatial relationship between immune cells and tumour cells can reflect the functional status of the immune microenvironment. In contrast, other immune cell subsets did not show a significant correlation between their spatial proximity and survival. This lack of correlation may be constrained by the sample size of the entire cohort; however, variations were noted among different molecular and pathological subtypes. This suggests that distinct MB subtypes may influence their unique microenvironment characteristics through the recruitment of differentiated immune cells and the remodelling of spatial patterns.^33^^,^^58^

While our study presents several important findings, it also has certain limitations: the possibility that mIF cut-points were data-driven and may not generalise without validation; and lack of functional assays to confirm mechanistic links. The patients in our cohort were all from a single centre in China, which may limit the global applicability of our results. Due to the retrospective nature of our study and the limited sample size, we were unable to assess somatic TP53 mutation status or MYC amplification using whole-exome sequencing (WES) or fluorescence in situ hybridization (FISH). As a result, molecular profiling data on these tumour-specific alterations were not available in our cohort. Validation in larger, multi-centre cohorts encompassing all molecular subtypes will enhance the robustness and generalisability of these findings. The inter-group comparison, illustrated by the boxplot, reveals limited differences between the groups, with most comparisons identified as not statistically significant. In future studies, we plan to supplement this information according to the WHO CNS5 (2021) guidelines, which will improve risk stratification for patients with MB. Another important limitation of this study is the utilization of a data-driven optimal threshold (surv_cutpoint) in the survival analysis. While this method is prevalent in exploratory biomarker research, it may result in inflated type I errors. Additionally, the validation cohort is based on RNA sequencing rather than multiplex immunofluorescence, which means that the absolute cut-off derived from the discovery cohort cannot be directly applied. Consequently, we opted to re-establish cohort-specific thresholds in the validation cohort. Despite the differences in exact thresholds, a consistent prognostic trend was observed across various platforms. This finding indicates a degree of robustness in our results; however, the definition of the threshold should still be regarded as exploratory. Further validation in larger, independent cohorts employing consistent detection methods is necessary to establish clinically applicable cutoffs.

In conclusion, our study provides a comprehensive overview of the immune cell landscape in MB and highlights its potential prognostic value. These findings contribute to our understanding of the immune mechanisms underlying MB and may guide future immunotherapy strategies. By further investigating the immune landscape and mechanisms in MB, we can identify new therapeutic targets and develop more effective treatments for MB, ultimately improving patient outcomes.

## Contributors

Conceptualization: M.C., X.S., T.S.; Methodology: M.C., X.S., Y.W.; Data Collection and Preparation: J.Z., J.G., X.G., Y.S., W.W., C.L., Y.T., Y.O., T.L., K.J.; Data Analysis and Visualization: M.C., X.S., J.Z.; Writing—Original Draft: M.C., X.S.; Writing—Review & Editing: M.D.T., X.L., L.Z., T.S.; Supervision: X.L., L.Z., T.S.

M.C., X.S., and T.S. accessed and verified the underlying data.

All authors have reviewed and approved the final version of the manuscript.

## Data sharing statement

The data supporting the findings of this study are available from the corresponding author upon request.

## Declaration of interests

M.C.—None declared; X.S.—Research Support: NNSFC, CPSF and CLS; Y.W.—None declared; J.Z.—None declared; J.G.—None declared; X.G.—None declared; Y.S.—None declared; W.W.—None declared; C.L.—None declared; Y.T.—None declared; Y.O.—None declared; T.L.—None declared; K.J.—None declared; M.D.T.—None declared; X.L.—None declared; L.Z.—Research Support: PWD & RPP-MRI; T.S.—None declared. The remaining authors declare no competing interests.

## Appendix ASupplementary data

Supplementary Materials

Supplemental Western blots
