# Supplementary figures and images for: Profiling tumour-infiltrating immune cells in a large paediatric medulloblastoma cohort: a retrospective analysis
Source: eBioMedicine. 2025 Nov 23;122:106043. doi: 10.1016/j.ebiom.2025.106043 (PMC12681837; doi:10.1016/j.ebiom.2025.106043)

Supplementary Figure 1

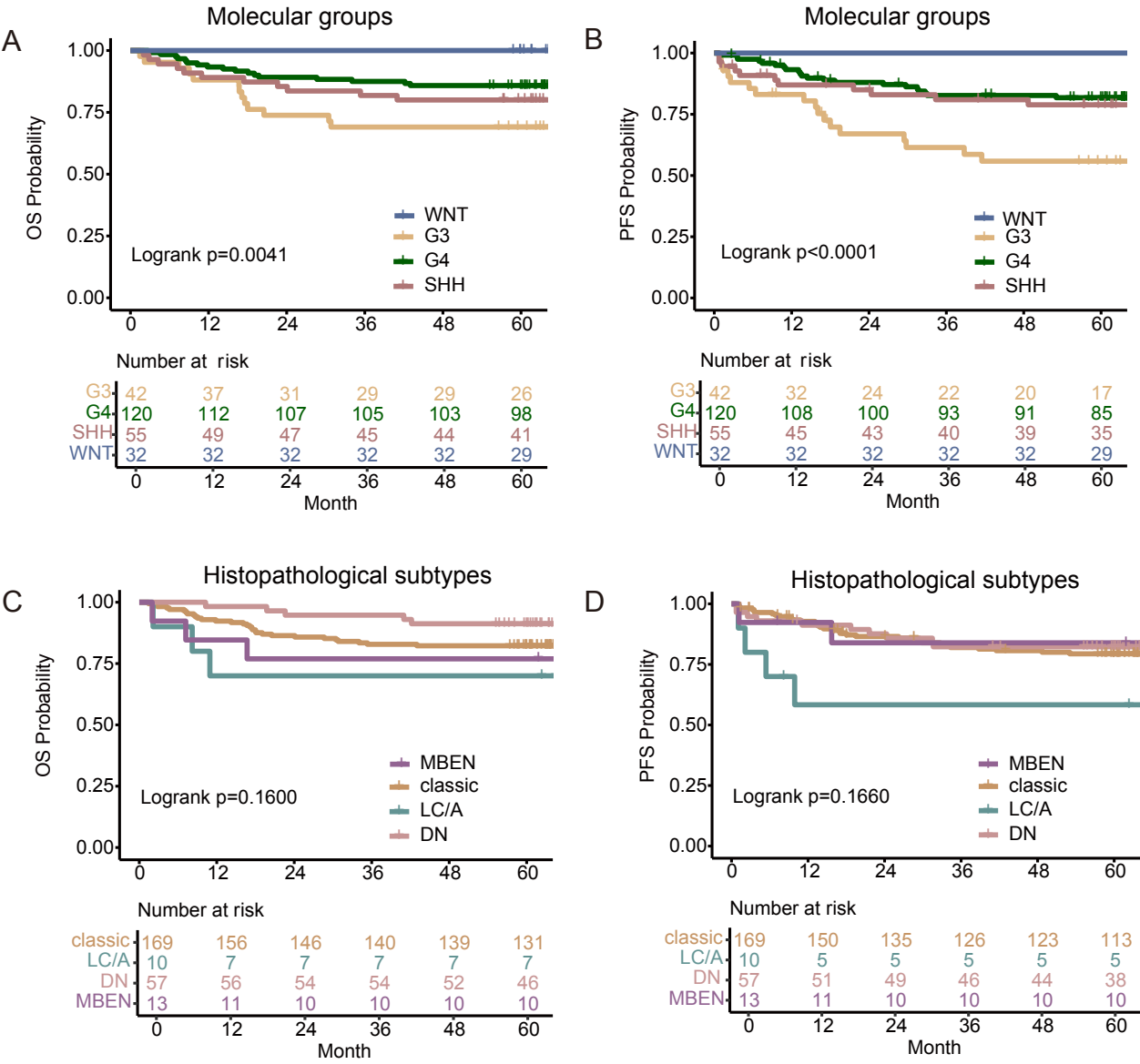

Supplementary Figure 2

A

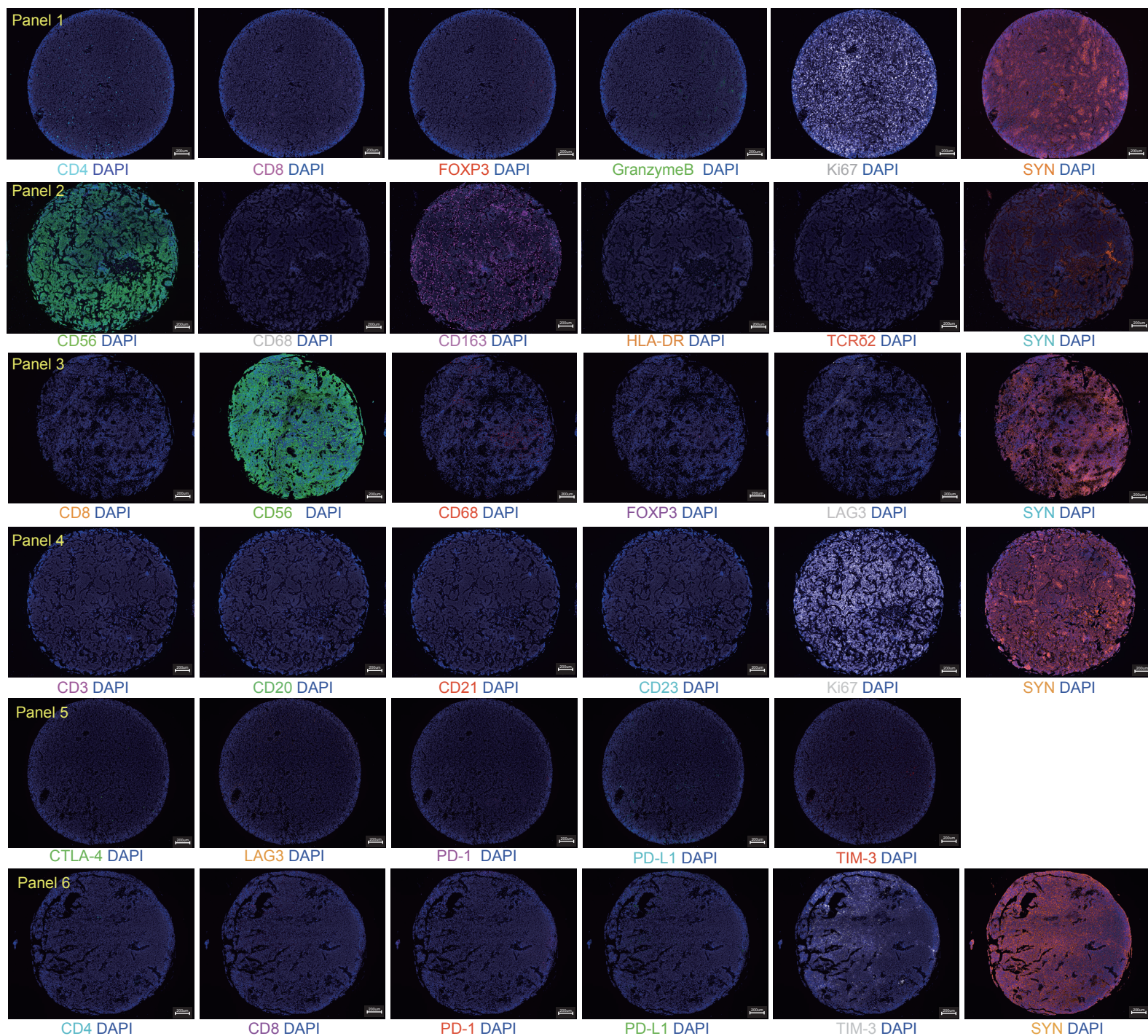

Supplementary Figure 3

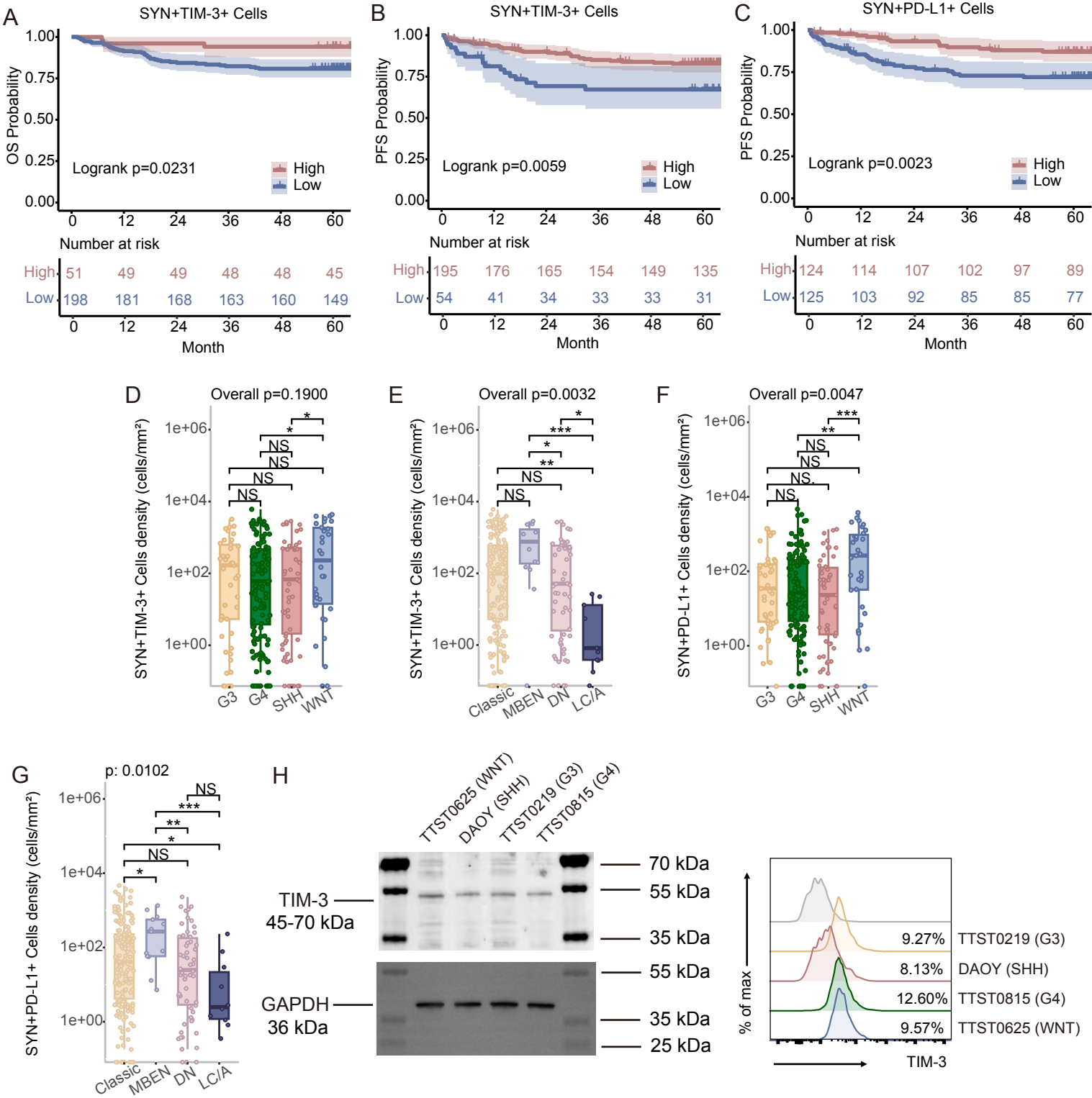

Supplement: Supplementary file 1 — Supplementary Figure [file mmc1.pdf]
