# Supplementary material for: Profiling tumour-infiltrating immune cells in a large paediatric medulloblastoma cohort: a retrospective analysis
Source: eBioMedicine. 2025 Nov 23;122:106043. doi: 10.1016/j.ebiom.2025.106043 (PMC12681837; doi:10.1016/j.ebiom.2025.106043)
Supplement: Supplementary file 2 — Supplementary Materials [file mmc2.docx]

**Supplementary Methods**

**Genome-wide Methylation Analysis**

The fresh consecutive testing set underwent DNA methylation classification for molecular diagnosis, and the specific procedure for DNA methylation classification is outlined in the following.^1^ The genomic DNA was meticulously extracted from formalin-fixed, paraffin-embedded (FFPE) specimens and subjected to bisulfite conversion employing the EZ DNA Methylation kit, in strict adherence to the recommended protocol provided by Zymo Research (Irvine, CA, USA). Subsequent to the conversion process, the DNA underwent amplification, fragmentation, Precipitation and rehydration of fragmented DNA, and was meticulously hybridized onto the Illumina Infinium MethylationEPIC v2.0 BeadChip array as per the specified guidelines of Illumina Inc (San Diego, CA). Data derived from methylation analyses were preprocessed utilizing the ChAMP package (version 1.28.4) within R Bioconductor (version 3.5.3). We adhered to the ChAMP standard pipeline for stringent quality control measures and normalization of the data. For the prediction of Medulloblastoma (MB) subgroups, we accessed a dedicated web-based platform that specializes in DNA methylation-based classification of central nervous system tumors available at [www.molecularneuropathology.org](http://www.molecularneuropathology.org/) (version 11b4).^2^

**mIF staining and cell density analysis**

The mIF staining was carried out using methods similar to those previously described and validated.^3^ Briefly, mIF staining was performed using an AlphaXTSA 7 kit (Alpha X Bio, CA). Before conducting the formal multiplex immunofluorescence experiments, we performed multiple single-plex tests for each antibody to optimize the staining conditions and ensure consistency across channels. All formal multiplex experiments were carried out by the same experienced operator using the same automated platform. The slices from the same panel were stained in the same batch. These measures effectively minimized batch variation, thereby eliminating the need for additional mathematical calibration. Various primary antibodies were applied sequentially, followed by incubation with horseradish peroxidase–conjugated secondary antibodies and tyramide signal amplification (TSA). The slides were placed in a microwave after each TSA step. The nuclei were stained with DAPI (Sigma–Aldrich, USA) after labeling all the human antigens. The entire staining process was completed using the AlphaXPainter X30 fully automated staining instrument. The antibodies used are detailed in the following six panels: Panel 1 included primary antibodies against CD4 (clone UMAB64, ZSGB-BIO), CD8 (clone SP16, ZSGB-BIO), FOXP3 (clone 236A/E7, Abcam), Granzyme B (clone EPR8260, Abcam) and Ki-67(clone UMAB107, ZSGB-BIO). Panel2 included primary antibodies against TCRδ2 (clone H4-1, Santa Cruz), CD56 (clone UMAB83, ZSGB-BIO), CD68 (clone KP1, ZSGB-BIO), CD163 (clone 10D6, ZSGB-BIO) and HLA-DR (clone SC06-78). Panel 3 included primary antibodies against PD-1 (clone UMAP199, ZSBG-BIO), PD-L1 (clone E1L3N, CST), TIM-3 (clone D5D5R, CST), CD4 (clone UMAB64, ZSGB-BIO) and CD8 (clone SP16, ZSGB-BIO). Panel 4 included primary antibodies against CD3(clone LN10, ZSBG-BIO), CD20 (clone EP459Y, Abcam), CD21 (clone 2G9, ZSBG-BIO), CD23 (clone EP75, ZSBG-BIO) and Ki-67 (clone UMAB107, ZSGB-BIO). Panel 5 included primary antibodies against PD-1(clone UMAP199, ZSBG-BIO), PD-L1 (clone E1L3N, CST), TIM-3 (clone D5D5R, CST), LAG-3 (clone EPR20261, Abcam) and CTLA-4 (clone UMAB249, ZSGB-BIO). Panel6 included primary antibodies against LAG-3 (clone EPR20261, Abcam), CD8 (clone SP16, ZSGB-BIO), CD56(clone UMAB83, ZSGB-BIO), CD68 (clone KP1, ZSGB-BIO) and FOXP3 (clone 236A/E7, Abcam). In all panels an anti-Synaptophysin (clone YE269, Abcam) antibody was used as tumor marker. The anti-mouse + rabbit Horseradish Peroxidase (HRP)-conjugated secondary antibody (Akoya Biosciences) was added for 10 minutes at room temperature and a different Tyramide Signal Amplification (TSA)-conjugated Opal fluorophore (Akoya Biosciences) was applied onto the tissues for 10 minutes. Then, the HIER step was performed, and for each cell marker the aforementioned steps were repeated in sequence. After seven sequential reactions, slides were counterstained with spectral DAPI (Akoya Biosciences) and mounted using Vectashield Hardset mounting medium (Vector Labs). All mIF stainings were validated using control tissue. The whole slide imaging system used ZEN 3.3 (ZEISS Axioscan 7; ZEISS, DE) for imaging, and data analysis was performed using Halo software (HALO 3.5; Indica Labs, USA) for quantitative statistics on individual cells. This included the delineation of tumor and stromal regions, various data type statistics for single and multiple biomarker combinations, as well as the counts of positive cells, positive rates, and positive cell density.

**Cell-to-cell distance analyses**

The spatial metrics between cells were determined using QuPath v0.3.0 image analysis software (the Queen's University of Belfast, Northern Ireland, UK) on the PanoATLAS workstation to recognition of cell morphology and spatial distribution. Whole slide fluorescence images were analysed with QuPath software The algorithm was constructed using Qupath through the steps of ticking, training, and confirmation. In algorithm construction, TUMOR, STROMA and TOTAL three tissue classifications were constructed, and the area and number of each tissue classification under the case were recorded. Accurate identification and counting of cells by Qupath software, and setting a reasonable threshold to identify positive cells, and counting the number of all cells with the number of positive cells. To evaluate the spatial relationships between immune cells and tumor cells, we conducted a spatial analysis to calculate the average number of immune cells distributed within a maximum 30 µm radius from the nuclear center of any given tumor cell.

**Characterization of immune cell phenotypes**

We generated cord plots to illustrate the interactions between cell phenotypes based on the co-expression of markers derived from each mIF panel. Moreover, we used uniform manifold approximation and projection (UMAP) to reduce dimensions and visualize the various immune cell phenotypes identified in each panel from the tumor. The findings were visualized using R Studio version 4.2.2 (<https://github.com/lmcinnes/umap>).

**Isolation and culture of primary MB cells**

During the surgical procedure, tumor tissue from the MB was aseptically collected and placed into a centrifuge tube containing DMEM (Dulbecco's Modified Eagle Medium; Gibco, Thermo Fisher Scientific). The tissue was washed twice with phosphate-buffered saline (PBS) to eliminate residual red blood cells. Using sterile scissors, the tissue was mechanically dissociated into fragments measuring 1-2 mm in diameter to prepare a single-cell suspension, followed by centrifugation at 1200 rpm for 3 minutes at 4°C. After centrifugation, 5 ml of DMEM, 100 µl of Collagenase Type IV powder, and 20 µl of DNase were added and mixed thoroughly. The mixture was then placed in a humidified incubator at 37°C with 5% CO2 for complete digestion for 15 minutes, with gentle pipetting every 5 minutes to ensure thorough contact. Following digestion, the suspension was filtered through a sterile 200 μm filter and centrifuged again at 1200 rpm for 3 minutes at 4°C. The cell pellet was subsequently resuspended in 5 ml of pre-prepared medium containing DMEM and the following additives: 10% fetal bovine serum, 1% N-2 supplement, 2% B-27 supplement, 20 ng/ml recombinant human fibroblast growth factor (Thermo Fisher), and 20 ng/ml recombinant human epidermal growth factor (R&D Systems). The cells were cultured in T25 culture flasks that had been pre-coated overnight with 2 ml of Matrigel in a humidified incubator at 37°C with 5% CO_2_. The culture conditions were maintained in a humidified incubator at 37°C with 5% CO_2_. The isolation and culture of primary cells were approved by the Ethics Committee of Beijing Tiantan Hospital (KY2014-021-02), with the patient's consent obtained for sample collection. For minors, informed consent was provided by their legal guardians (parents or other guardians) on their behalf.

**Western blot and flow cytometry detection**

Primary MB tumor cells (TTST2019, TTST0815, and TTST0625) and the DAOY cell line were lysed in RIPA buffer and subjected to protein lysis at 100°C for 5 min in a metal bath. The samples were then centrifuged at 13,000 rpm and 4°C for 15 min. The protein concentrations were measured, and the lysates were separated using 12% SDS-PAGE, followed by standard protocols for Western blotting. TIM-3 antibody (45208; CST, USA) was used to detect the target protein, along with the internal control anti-GAPDH antibody (ab9484; Abcam, UK). For flow cytometry analysis, the dissociated MB tumor cells were stained with TIM-3-PE (364805; Biolegend, USA) antibody or isotype control. The stained cells were analyzed using a CytoFlex flow cytometer (Beckman, USA) after a 30-min incubation on ice in the dark.

**Supplement references**

1. Wang YJ, Wang P, Yan Z, et al: Advancing presurgical non-invasive molecular subgroup prediction in medulloblastoma using artificial intelligence and MRI signatures. Cancer Cell 42:1239-1257.e7, 2024.

2. Capper D, Jones DTW, Sill M, et al: DNA methylation-based classification of central nervous system tumours. Nature 555:469-474, 2018.

3. Viratham Pulsawatdi, A. et al. A robust multiplex immunofluorescence and digital pathology workflow for the characterisation of the tumour immune microenvironment. Molecular oncology, 2020,14, 2384-2402.

4. Hidehiro Takei, Meenakshi B Bhattacharjee. New immunohistochemical markers in the evaluation of central nervous system tumors: a review of 7 selected adult and pediatric brain tumors. Arch Pathol Lab Med. 2007,131(2):234-41.

**Supplementary Table 1. Reagent and resource**

| Panel | Reagent | Identifier | Source | IDENTIFIER |
| --- | --- | --- | --- | --- |
| Panel1 | CD4 | clone UMAB64 | ZSGB-BIO | Cat# ZA-0519; RRID: AB_3076264 |
|  | CD8 | clone SP16 | ZSGB-BIO | Cat# ZA-0508; RRID: AB_2890107 |
|  | FOXP3 | clone 236A/E7 | Abcam | Cat# 3100-1; RRID: AB_2104897 |
|  | Granzyme B | clone EPR8260 | Abcam | Cat# ab3654; RRID: AB_303979 |
|  | Ki-67 | clone UMAB107 | ZSGB-BIO | Cat# ZM-0165; RRID: AB_2636802 |
|  | Synaptophysin | clone YE269 | Abcam | Cat# 1870-1; RRID: AB_765072 |
| Panel2 | TCRδ2 | clone H4-1 | Santa Cruz | Cat# sc-100289; RRID: AB_1130061 |
|  | CD56 | clone UMAB83 | ZSGB-BIO | Cat# ZM-0057; RRID: AB_2890109 |
|  | CD68 | clone KP1 | ZSGB-BIO | Cat# ZM-0464; RRID: AB_3076266 |
|  | CD163 | clone 10D6 | ZSGB-BIO | Cat# ZM-0428, RRID: AB_3714707 |
|  | HLA-DR | clone SC06-78 | HUABIO | Cat# R1510-34; RRID: AB_3073342 |
|  | Synaptophysin | clone YE269 | Abcam | Cat# 1870-1; RRID: AB_765072 |
| Panel3 | PD-1 | clone UMAP199 | ZSGB-BIO | Cat# ZM-0381; RRID: AB_2921363 |
|  | PD-L1 | clone E1L3N | CST | Cat# 13684; RRID: AB_2864409 |
|  | TIM-3 | clone D5D5R | CST | Cat# 83882; RRID: AB_2800033 |
|  | CD4 | clone UMAB64 | ZSGB-BIO | Cat# ZA-0519; RRID: AB_3076264 |
|  | CD8 | clone SP16 | ZSGB-BIO | Cat# ZA-0508; RRID: AB_2890107 |
|  | Synaptophysin | clone YE269 | Abcam | Cat# 1870-1; RRID: AB_765072 |
| Panel4 | CD3 | clone LN10 | ZSGB-BIO | Cat# ZM-0417; RRID: AB_2890105 |
|  | CD20 | clone EP459Y | Abcam | Cat# AP-0012; RRID: AB_10703983 |
|  | CD21 | clone 2G9 | ZSGB-BIO | Cat# ZM-0040, RRID: AB_3714709 |
|  | CD23 | clone EP75 | ZSGB-BIO | Cat# ZA-0516, RRID: AB_3714710 |
|  | Ki-67 | clone UMAB107 | ZSGB-BIO | Cat# ZM-0165; RRID: AB_2636802 |
|  | Synaptophysin | clone YE269 | Abcam | Cat# 1870-1; RRID: AB_765072 |
| Panel5 | PD-1 | clone UMAP199 | ZSGB-BIO | Cat# ZM-0381; RRID: AB_2921363 |
|  | PD-L1 | clone E1L3N | CST | Cat# 13684; RRID: AB_2864409 |
|  | TIM-3 | clone D5D5R | CST | Cat# 83882; RRID: AB_2800033 |
|  | LAG-3 | clone EPR20261 | Abcam | Cat# ab209236; RRID: AB_2883982 |
|  | CTLA-4 | clone UMAB249 | ZSGB-BIO | Cat# ZM-0035, RRID: AB_3714706 |
| Panel6 | LAG-3 | clone EPR20261 | Abcam | Cat# ab209236; RRID: AB_2883982 |
|  | CD8 | clone SP16 | ZSGB-BIO | Cat# ZA-0508; RRID: AB_2890107 |
|  | CD56 | clone UMAB83 | ZSGB-BIO | Cat# ZM-0057; RRID: AB_2890109 |
|  | CD68 | clone KP1 | ZSGB-BIO | Cat# ZM-0464; RRID: AB_3076266 |
|  | FOXP3 | clone 236A/E7 | Abcam | Cat# 3100-1; RRID: AB_2104897 |
|  | Synaptophysin | clone YE269 | Abcam | Cat# 1870-1; RRID: AB_765072 |
| WB | TIM-3 | clone D5D5R | CST | Cat# 83882; RRID: AB_2800033 |
| Flow | TIM-3 | Tim3-PE | Biolegend | Cat# 345006; RRID: AB_2116576 |
| Cell Line | DAOY | NA | ATCC | RRID: CVCL_1167 |
|  | TTYY0625 | NA | This study | NA |
|  | TTYY0219 | NA | This study | NA |
|  | TTYY0815 | NA | This study | NA |

**Supplementary Table 2. Phenotypes frequently observed in the six multiplex immunofluorescence panels of the MB cohort**

| Panel | Marker co-expression | Phenotype |
| --- | --- | --- |
| 1 | CD4+ | Th cells |
|  | CD4+FOXP3+ | Regulatory T-cells |
|  | CD4+Ki67+ | Th cells expressing Ki67 |
|  | CD8+ | Cytotoxic T-cells |
|  | CD8+Granzyme B+ | Activated cytotoxic T-cells |
|  | CD8+Ki67+ | Cytotoxic T-cells expressing Ki67 |
|  | SYN+ | Medulloblastoma |
|  | SYN+Ki67+ | Medulloblastoma expressing Ki67 |
| 2 | CD68+ | Tumor-associated macrophages |
|  | CD68+CD163-HLA-DR+ | Type I tumor-associated macrophages |
|  | CD68+CD163+HLA-DR- | Type II tumor-associated macrophages |
|  | CD68+CD163+HLA-DR+ | Mixed tumor-associated macrophages |
|  | CD56+SYN- | NK cells |
|  | TCRδ2 | γδT cells |
| 3 | CD8+LAG-3+ | Cytotoxic T-cells expressing LAG-3 |
|  | CD68+LAG-3+ | Macrophages expressing LAG-3 |
|  | CD56+LAG-3+ | NK cells expressing LAG-3 |
|  | FOXP3+LAG-3 | Treg cells expressing LAG-3 |
|  | SYN+LAG-3+ | Medulloblastoma expressing LAG-3 |
| 4 | CD3+ | All T lymphocytes |
|  | CD20+CD21-CD23- | Early tertiary lymphoid structures |
|  | CD20+CD21+CD23- | Primary tertiary lymphoid structures |
|  | CD20+CD21+CD23+ | Secondary tertiary lymphoid structures |
| 5 | PD-1+ | Programmed cell death protein 1 |
|  | TIM-3+ | T cell immunoglobulin-3 |
|  | LAG-3+ | Lymphocyte-activation gene 3 |
|  | CTLA-4+ | Cytotoxic T-lymphocyte-associated protein 4 |
|  | PD-L1+ | Programmed cell death 1 ligand 1 |
| 6 | CD4+TIM-3+ | Th cells expressing TIM-3 |
|  | CD4+PD-1+ | Th cells expressing PD-1 |
|  | CD8+PD-1+ | Cytotoxic T-cells expressing PD-1 |
|  | CD8+TIM-3+ | Cytotoxic T-cells expressing TIM-3 |
|  | SYN+PD-1+ | Medulloblastoma expressing PD-1 |
|  | SYN+TIM-3+ | Medulloblastoma expressing TIM-3 |
|  | SYN+PD-L1+ | Medulloblastoma expressing PD-L1 |

SYN, synaptophysin.

**Supplementary Table 3. Histopathological subtypes of the 249 patients with pediatric MB whose tumor specimens were included in our study**

| Characteristic | All patients | Medulloblastoma | | | |  |
| --- | --- | --- | --- | --- | --- | --- |
|  |  | classic | DN | LC/A | MBEN | *p* value |
| Frequency N (%) | 249 | 169  (67.871%) | 57  (22.892%) | 10  (4.016%) | 13  (5.221%) |  |
| Age |  |  |  |  |  |  |
| Mean (SD) | 7.448(3.394) | 7.372(3.389) | 8.242(3.420) | 6.714(3.200) | 5.889(3.257) | 0.0256^*^ |
| Median (Q1, Q3) | 7(5, 10) | 7(5, 10) | 8(6, 11) | 7(5, 8) | 7(3, 8) |  |
| Sex |  |  |  |  |  |  |
| M | 167  (67.068%) | 108  (64.671%) | 41  (24.551%) | 7  (4.192%) | 11  (6.587%) | 0.3580 |
| F | 82(32.932%) | 61(74.390%) | 16(19.512%) | 3(3.659%) | 2(2.439%) |  |
| Postoperative residual |  |  |  |  |  |  |
| <1.5cm^2^ | 249  (100.000%) | 169  (67.871%) | 57  (22.892%) | 10  (4.016%) | 13  (5.221%) |  |
| ≥1.5cm^2^ | 0(0.000%) | 0(0.000%) | 0(0.000%) | 0(0.000%) | 0(0.000%) |  |
| Total | 249  (100.000%) | 169  (67.871%) | 57  (22.892%) | 10  (4.016%) | 13  (5.221%) |  |
| Radiotherapy |  |  |  |  |  |  |
| Y | 248  (99.598%) | 168  (67.742%) | 57  (22.984%) | 10  (4.032%) | 13  (5.242%) | 0.9243 |
| N | 1(0.402%) | 1(100.000%) | 0(0.000%) | 0(0.000%) | 0(0.000%) |  |
| Total | 249  (100.000%) | 169  (67.871%) | 57  (22.892%) | 10  (4.016%) | 13  (5.221%) |  |
| Chemotherapy |  |  |  |  |  |  |
| Y | 248  (99.598%) | 168  (67.742%) | 57  (22.984%) | 10  (4.032%) | 13  (5.242%) | 0.9243 |
| N | 1(0.402%) | 1(100.000%) | 0(0.000%) | 0(0.000%) | 0(0.000%) |  |
| Total | 249  (100.000%) | 169  (67.871%) | 57  (22.892%) | 10  (4.016%) | 13  (5.221%) |  |
| Histological Stubtypes |  |  |  |  |  |  |
| WNT | 32(12.851%) | 28(87.500%) | 1(3.125%) | 3(9.375%) | 0(0.000%) | 0.000^****^ |
| SHH | 55(22.088%) | 6(10.909%) | 33(60.000%) | 3(5.455%) | 13(23.636%) |  |
| G3 | 42(16.867%) | 35(83.333%) | 3(7.143%) | 4(9.524%) | 0(0.000%) |  |
| G4 | 120  (48.193%) | 100  (83.333%) | 20  (16.667%) | 0  (0.000%) | 0  (0.000%) |  |
| Survival Status |  |  |  |  |  |  |
| Alive | 207  (83.133%) | 138  (66.667%) | 52  (25.121%) | 7  (3.382%) | 10  (4.831%) | 0.2111 |
| Decease | 42  (16.867%) | 31  (73.810%) | 5  (11.905%) | 3  (7.143%) | 3  (7.143%) |  |

* *p* <0.0500, ** *p* <0.0100, *** *p* <0.0010, **** *p* <0.0001. The statistical analysis involved chi-square tests to determine p values for categorical variables. Fisher’s exact test was employed specifically for categorical variables where expected counts were below five. For continuous data, comparisons of mean values were conducted using ANOVA tests.

**Supplementary Table 4.** **Clinical risk of MB patients**

| Characteristic (age) | All | <3 years | ≥3 years | *p* value |
| --- | --- | --- | --- | --- |
| Frequency N（%） | 249 | 30 (12.048%) | 219 (87.952%) |  |
| Sex |  |  |  | 0.0082 |
| M | 167 (67.068%) | 27 (16.168%) | 140 (83.832%) |  |
| F | 82 (32.932%) | 3 (3.659%) | 79 (96.341%) |  |
| Total | 249(100.000%) | 30 (12.048%) | 219 (87.852%) |  |
| M staging |  |  |  | 1.0000 |
| M0 | 211 (84.739%) | 25 (11.848%) | 186 (88.152%) |  |
| M+ | 38 (15.261%) | 5 (13.158%) | 33 (86.842%) |  |
| Total | 249(100.000%) | 30 (12.048%) | 219 (87.852%) |  |
| Postoperative residual |  |  |  |  |
| <1.5cm^2^ | 249(100.000%) | 30 (12.048%) | 219 (87.852%) |  |
| ≥1.5cm^2^ | 0(0.000%) | 0(0.000%) | 0(0.000%) |  |
| Total | 249(100.000%) | 30 (12.048%) | 219 (87.852%) |  |
| Histopathological subtype |  |  |  | 0.1490 |
| DN | 57 (22.892%) | 6 (10.526%) | 51 (89.474%) |  |
| Classic | 169 (67.871%) | 18 (10.651%) | 151 (89.349%) |  |
| LC/A | 10 (4.016%) | 2 (20.000%) | 8 (80.000%) |  |
| MBEN | 13 (5.221%) | 4 (30.769%) | 9 (69.231%) |  |
| Total | 249(100.000%) | 30 (12.048%) | 219 (87.852%) |  |
| Clinical risk |  |  |  | 0.0000^****^ |
| High-risk | 83 (33.333%) | 21 (25.301%) | 62 (74.699%) |  |
| Average-risk | 166 (66.667%) | 9 (5.422%) | 157 (94.578%) |  |
| Total | 249(100.000%) | 30 (12.048%) | 219 (87.852%) |  |
| Survival Status |  |  |  |  |
| High-risk |  |  |  | 1.0000 |
| Alive | 57 (68.675%) | 14 (24.561%) | 43 (75.439%) |  |
| Decease | 26 (31.325%) | 7 (26.923%) | 19 (73.077%) |  |
| Total | 83 | 21 (25.301%) | 62 (74.699%) |  |
| Average-risk |  |  |  | 1.0000 |
| Alive | 150 (90.361%) | 8 (5.333%) | 142 (94.667%) |  |
| Decease | 16 (9.639%) | 1 (6.250%) | 15 (93.750%) |  |
| Total | 166 | 9 (5.422%) | 157 (94.578%) |  |
| Overall Survival Rate |  |  |  |  |
| High-risk | 68.675% | 66.667% | 69.355% |  |
| Average-risk | 90.361% | 88.889% | 90.446% |  |

* *p* <0.0500, ** *p* <0.0100, *** *p* <0.0010, **** *p* <0.0001. The statistical analysis involved chi-square tests to determine p values for categorical variables. Fisher’s exact test was employed specifically for categorical variables where expected counts were below five. For continuous data, comparisons of mean values were conducted using ANOVA tests.

**Supplementary Table 5. Compare the differences in the density of immune cells across the histopathological subtypes and molecular groups**

| Variable (cells) | *p* value | |
| --- | --- | --- |
|  | Histopathological subtypes | Molecular groups |
| CD3+ | 0.0073 | **0.0312** |
| CD4+ | 0.6380 | **0.5050** |
| CD4+FOXP3+ | 0.6190 | 0.9550 |
| CD4+Ki67+ | 0.5660 | **0.0067** |
| CD8+ | 0.0529 | 0.3560 |
| CD8+GranzymeB+ | **0.0377** | 0.9090 |
| CD8+Ki67+ | 0.1540 | **0.0319** |
| CD20+ | 0.9780 | **0.0010** |
| CD56+ | **0.0485** | **<0.0001** |
| CD56+LAG-3+ | **0.0220** | **<0.0001** |
| CD56+SYN- | 0.7060 | **<0.0001** |
| CD68+ | 0.9780 | 0.3920 |
| CD68+CD163-HLA-DR+ | 0.1700 | 0.7000 |
| CD68+CD163+HLA-DR- | 0.3490 | **0.0099** |
| CD68+CD163+HLA-DR+ | 0.5270 | 0.4440 |
| Ki67+CD3+ | **0.0053** | **0.0412** |
| Ki67+SYN+ | 0.8920 | **0.0114** |
| CD8+ LAG-3+ | **0.0210** | **0.0003** |
| CD68+ LAG-3+ | 0.8160 | 0.2330 |
| FOXP3+ LAG-3+ | 0.1860 | **<0.0001** |
| PD-1+ | 0.0821 | 0.5040 |
| PD-L1+ | 0.5040 | 0.7550 |
| LAG-3+ | 0.2370 | 0.1780 |
| TIM-3+ | 0.3890 | 0.1700 |
| CTLA-4+ | 0.4010 | 0.0671 |
| CD4+PD-1+ | 0.3620 | 0.1840 |
| CD8+PD-1+ | 0.3220 | 0.0999 |
| SYN+PD-L1+ | **0.0102** | **0.0047** |
| TCRδ2 | 0.4940 | **0.0003** |
| TIM3+CD4+ | 0.4780 | 0.0954 |
| TIM3+CD8+ | **0.0026** | 0.2330 |

**Supplementary Table 6. Pairwise comparison of the differences in immune cell density across the molecular groups**

| marker | *p^a^* value | | | | | |
| --- | --- | --- | --- | --- | --- | --- |
|  | *G3 vs G4* | *G3 vs SHH* | *G3 vs WNT* | *G4 vs SHH* | *G4 vs WNT* | *SHH vs WNT* |
| CD3+ | 1.0000 | 1.0000 | 0.2110 | 0.7960 | 0.0320 | 1.0000 |
| CD4+ | 1.0000 | 1.0000 | 1.0000 | 0.8280 | 1.0000 | 1.0000 |
| CD4+FOXP3+ | 1.0000 | 1.0000 | 1.0000 | 1.0000 | 1.0000 | 1.0000 |
| CD4+Ki67+ | 1.0000 | 0.1050 | 0.6170 | 0.0120 | 0.2730 | 1.0000 |
| CD8+ | 1.0000 | 1.0000 | 1.0000 | 1.0000 | 0.4370 | 1.0000 |
| CD8+GranzymeB+ | 1.0000 | 1.0000 | 1.0000 | 1.0000 | 1.0000 | 1.0000 |
| CD8+Ki67+ | 1.0000 | 1.0000 | 0.1710 | 1.0000 | 0.0210 | 0.0930 |
| CD20+ | 1.0000 | 1.0000 | 0.0090 | 1.0000 | 0.0010 | 0.0030 |
| CD56+LAG-3+ | 0.0000 | 0.0020 | 1.0000 | 1.0000 | 0.0020 | 0.0040 |
| CD56+SYN- | 1.0000 | 0.0040 | 0.1740 | 0.4190 | 0.0009 | 0.0000 |
| CD68+ | 1.0000 | 1.0000 | 0.8220 | 1.0000 | 0.8220 | 0.7030 |
| CD68+CD163-HLA-DR+ | 1.0000 | 1.0000 | 1.0000 | 1.0000 | 1.0000 | 1.0000 |
| CD68+CD163+HLA-DR- | 1.0000 | 1.0000 | 0.3290 | 0.3420 | 0.0090 | 0.8830 |
| CD68+CD163+HLA-DR+ | 1.0000 | 1.0000 | 1.0000 | 0.7910 | 1.0000 | 1.0000 |
| CD3+Ki67+ | 1.0000 | 1.0000 | 0.1730 | 1.0000 | 0.0540 | 1.0000 |
| SYN+Ki67+ | 1.0000 | 1.0000 | 0.4980 | 0.1840 | 0.0160 | 1.0000 |
| CD8+LAG-3+ | 0.0080 | 0.0170 | 1.0000 | 1.0000 | 0.0310 | 0.0460 |
| CD68+LAG-3+ | 1.0000 | 0.8650 | 1.0000 | 1.0000 | 0.8500 | 0.4470 |
| FOXP3+LAG-3+ | 0.0020 | 0.0310 | 1.0000 | 1.0000 | 0.0020 | 0.0190 |
| PD-1+ | 1.0000 | 1.0000 | 1.0000 | 0.8510 | 1.0000 | 1.0000 |
| PD-L1+ | 1.0000 | 1.0000 | 1.0000 | 1.0000 | 1.0000 | 1.0000 |
| LAG-3+ | 1.0000 | 1.0000 | 1.0000 | 1.0000 | 1.0000 | 1.0000 |
| TIM-3+ | 1.0000 | 0.7970 | 1.0000 | 0.0401 | 1.0000 | 1.0000 |
| CTLA-4+ | 1.0000 | 1.0000 | 0.7620 | 0.1770 | 0.0426 | 1.0000 |
| CD4+PD-1+ | 1.0000 | 1.0000 | 0.3280 | 1.0000 | 0.7760 | 0.2860 |
| CD8+PD-1+ | 1.0000 | 1.0000 | 0.1140 | 1.0000 | 0.1790 | 0.3090 |
| SYN+PD-L1+ | 1.0000 | 1.0000 | 0.1750 | 0.1600 | 0.0082 | 0.0008 |
| TCRδ2+ | 0.0710 | 0.6750 | 0.9750 | 1.0000 | 0.0010 | 0.0200 |
| CD4+TIM-3+ | 1.0000 | 1.0000 | 0.3180 | 1.0000 | 0.1080 | 1.0000 |
| CD8+TIM-3+ | 1.0000 | 1.0000 | 0.9100 | 1.0000 | 0.2840 | 0.4180 |

a: The significance values have been adjusted using the Bonferroni correction method for multiple tests.

**Supplementary Table 7. Pairwise comparison of the differences in immune cell density across the histopathological subtypes**

| marker | *p^a^* value | | | | | |
| --- | --- | --- | --- | --- | --- | --- |
|  | *Classic vs MBEN* | *Classic vs DN* | *Classic vs LC/A* | *MBEN vs DN* | *MBEN vs LC/A* | *DN vs LC/A* |
| CD3+ | 0.1540 | 1.0000 | 0.0870 | 0.0091 | 0.0004 | 0.3300 |
| CD4+ | 1.0000 | 1.0000 | 1.0000 | 1.0000 | 1.0000 | 1.0000 |
| CD4+FOXP3+ | 1.0000 | 1.0000 | 1.0000 | 1.0000 | 1.0000 | 1.0000 |
| CD4+Ki67+ | 1.0000 | 1.0000 | 1.0000 | 1.0000 | 1.0000 | 1.0000 |
| CD8+ | 0.5850 | 1.0000 | 0.2370 | 1.0000 | 0.0380 | 0.1830 |
| CD8+GranzymeB+ | 0.0400 | 1.0000 | 1.0000 | 0.1450 | 0.0760 | 1.0000 |
| CD8+Ki67+ | 0.7200 | 1.0000 | 0.7300 | 0.6380 | 0.1420 | 1.0000 |
| CD20+ | 1.0000 | 1.0000 | 1.0000 | 1.0000 | 1.0000 | 1.0000 |
| CD56+LAG-3+ | 1.0000 | 0.9480 | 0.1590 | 1.0000 | 0.0550 | 0.0370 |
| CD56+SYN- | 1.0000 | 1.0000 | 1.0000 | 1.0000 | 1.0000 | 1.0000 |
| CD68+ | 1.0000 | 1.0000 | 1.0000 | 1.0000 | 1.0000 | 1.0000 |
| CD68+CD163-HLA-DR+ | 1.0000 | 0.2210 | 1.0000 | 1.0000 | 1.0000 | 1.0000 |
| CD68+CD163+HLA-DR- | 1.0000 | 0.6250 | 1.0000 | 1.0000 | 1.0000 | 1.0000 |
| CD68+CD163+HLA-DR+ | 1.0000 | 1.0000 | 1.0000 | 1.0000 | 1.0000 | 1.0000 |
| CD3+ Ki67+ | 0.0440 | 1.0000 | 0.1960 | 0.0400 | 0.0030 | 0.3870 |
| SYN+Ki67+ | 1.0000 | 1.0000 | 1.0000 | 1.0000 | 1.0000 | 1.0000 |
| CD8+LAG-3+ | 1.0000 | 0.1980 | 0.6800 | 1.0000 | 0.3410 | 0.0840 |
| CD68+LAG-3+ | 1.0000 | 1.0000 | 1.0000 | 1.0000 | 1.0000 | 1.0000 |
| FOXP3+LAG-3+ | 1.0000 | 1.0000 | 0.3510 | 1.0000 | 0.7560 | 0.1990 |
| PD-1+ | 0.0260 | 1.0000 | 1.0000 | 0.1210 | 0.0260 | 0.9360 |
| PD-L1+ | 1.0000 | 1.0000 | 1.0000 | 1.0000 | 0.8830 | 1.0000 |
| LAG-3+ | 1.0000 | 1.0000 | 0.2350 | 1.0000 | 1.0000 | 0.1990 |
| TIM-3+ | 1.0000 | 1.0000 | 1.0000 | 1.0000 | 1.0000 | 0.6380 |
| CTLA-4+ | 1.0000 | 1.0000 | 0.5110 | 1.0000 | 1.0000 | 0.8350 |
| CD4+PD-1+ | 1.0000 | 1.0000 | 0.7310 | 1.0000 | 1.0000 | 1.0000 |
| CD8+PD-1+ | 0.8980 | 1.0000 | 1.0000 | 1.0000 | 1.0000 | 1.0000 |
| SYN+PD-L1+ | 0.1320 | 1.0000 | 0.2580 | 0.0089 | 0.0009 | 0.8930 |
| TCRδ2+ | 1.0000 | 1.0000 | 0.9440 | 1.0000 | 0.8580 | 1.0000 |
| CD4+TIM-3+ | 1.0000 | 1.0000 | 1.0000 | 0.9390 | 1.0000 | 1.0000 |
| CD8+TIM-3+ | 0.1160 | 1.0000 | 0.0560 | 0.0390 | 0.0020 | 0.2770 |

a: The significance values have been adjusted using the Bonferroni correction method for multiple tests.

**Supplementary Table 8. Comparison of the cell numbers within a mean distance of 30 μm from the SYN+ to the immune cells across molecular groups**

| Variable (mean cells) | WNT | SHH | Group3 | Group4 | *p value* |
| --- | --- | --- | --- | --- | --- |
| SYN+ to CD4+ | 25.815 | 21.927 | 24.837 | 21.433 | 0.4020 |
| SYN+ to CD4+FOXP3+ | 5.496 | 19.323 | 13.955 | 10.520 | 0.1150 |
| SYN+ to CD4+Ki67+ | 12.881 | 22.325 | 12.968 | 13.962 | 0.5070 |
| SYN+ to CD56+SYN- | 13.118 | 18.089 | 19.938 | 21.765 | 0.8540 |
| SYN+ to CD8+ | 10.366 | 17.756 | 20.253 | 14.481 | 0.5830 |
| SYN+ to CD8+GranzymeB+ | 4.419 | 3.0186 | 4.701 | 2.546 | 0.8920 |
| SYN+ to CD68+CD163+ HLADR- | 24.847 | 17.868 | 11.543 | 10.318 | 0.5190 |
| SYN+ to CD68+CD163- HLADR+ | 0 | 3.224 | 2.306 | 3.825 | 0.8130 |
| SYN+ to CD68+CD163+ HLADR+ | 0 | 10.076 | 3.017 | 3.590 | 0.2820 |
| SYN+ to CD8+Ki67+ | 10.541 | 10.489 | 17.875 | 8.594 | 0.3970 |
| SYN+ to TCRδ2+ | 19.776 | 7.569 | 17.882 | 11.363 | 0.3970 |
| SYN+ to CD4+PD-1+ | 25.816 | 21.927 | 24.837 | 21.433 | 0.4020 |
| SYN+ to CD4+PD-1+TIM-3+ | 7.481 | 3.431 | 5.036 | 5.450 | 0.4710 |
| SYN+ to CD4+TIM-3+ | 20.319 | 12.032 | 15.010 | 12.745 | 0.0981 |
| SYN+ to CD56+LAG-3+ | 15.972 | 16.198 | 13.454 | 11.888 | 0.2411 |
| SYN+ to CD68+LAG-3+ | 4.172 | 13.291 | 2.723 | 11.177 | **<0.0001** |
| SYN+ to CD8+LAG-3+ | 0 | 2.586 | 0 | 1.046 | 0.0670 |
| SYN+ to FOXP3+LAG-3+ | 2.447 | 7.753 | 0.742 | 7.795 | **0.0008** |
| SYN+ to CD8+PD-1+ | 0.819 | 8.769 | 1.401 | 8.590 | **<0.0001** |
| SYN+ to CD8+PD-1+ TIM-3+ | 6.131 | 2.953 | 1.313 | 2.642 | 0.0950 |
| SYN+ to CD8+TIM-3+ | 17.619 | 14.321 | 14.891 | 14.218 | 0.9890 |

**Supplementary Table 9.** **Comparison of the cell numbers within a mean distance of 30 μm from the SYN+ to the immune cells across histopathological subtypes**

| Variable (mean cells) | Classic | DN | LC/A | MBEN | *p value* |
| --- | --- | --- | --- | --- | --- |
| SYN+ to CD4+ | 24.369 | 16.774 | 25.477 | 25.666 | 0.3072 |
| SYN+ to CD4+FOXP3+ | 12.163 | 11.482 | 13.051 | 26.519 | 0.6089 |
| SYN+ to CD4+Ki67+ | 15.085 | 11.553 | 25.711 | 24.976 | 0.7531 |
| SYN+ to CD56+SYN- | 20.273 | 19.626 | 12.914 | 24.908 | 0.9191 |
| SYN+ to CD8+ | 15.626 | 16.365 | 12.723 | 25.632 | 0.7030 |
| SYN+ to CD8+GranzymeB+ | 3.784 | 2.932 | 0 | 0 | 0.8970 |
| SYN+ to CD68+CD163+ HLADR- | 11.993 | 13.865 | 26.6912 | 24.095 | 0.3510 |
| SYN+ to CD68+CD163- HLADR+ | 1.812 | 5.168 | 10.375 | 0 | 0.3293 |
| SYN+ to CD68+CD163+ HLADR+ | 1.703 | 5.228 | 26.390 | 22.147 | **0.0006** |
| SYN+ to CD8+Ki67+ | 12.291 | 5.897 | 12.829 | 25.586 | 0.4390 |
| SYN+ to TCRδ2+ | 12.800 | 13.744 | 25.833 | 0 | 0.2830 |
| SYN+ to CD4+PD-1+ | 5.572 | 4.628 | 3.241 | 4.407 | 0.8720 |
| SYN+ to CD4+PD-1+TIM-3+ | 2.758 | 2.780 | 0 | 3.974 | 0.7350 |
| SYN+ to CD4+TIM-3+ | 13.827 | 12.500 | 4.532 | 19.672 | 0.2190 |
| SYN+ to CD56+LAG-3+ | 8.449 | 11.150 | 0 | 17.889 | **0.0024** |
| SYN+ to CD68+LAG-3+ | 0.592 | 2.382 | 0 | 2.094 | 0.1480 |
| SYN+ to CD8+LAG-3+ | 5.332 | 7.491 | 0 | 10.379 | 0.0971 |
| SYN+ to FOXP3+LAG-3+ | 6.039 | 8.115 | 0 | 8.478 | 0.2030 |
| SYN+ to CD8+PD-1+ | 3.222 | 3.331 | 0 | 0 | 0.3790 |
| SYN+ to CD8+ PD-1+TIM-3+ | 3.222 | 2.807 | 0 | 0 | 0.3810 |
| SYN+ to CD8+TIM-3+ | 14.719 | 12.929 | 3.408 | 22.175 | **0.0060** |

**Supplementary Table 10. Multivariate Cox proportional hazards regression (*high vs low*) model for OS in MB (n=249)**

| **Variable** | **HR (95%CI)** | ***p value*** | **Ph *p* biomarker** | **Ph *p* global** |
| --- | --- | --- | --- | --- |
| CD4+ | 0.47 (0.25-0.89) | **0.0207** | 0.4400 | 0.1120 |
| CD8+ | 0.90 (0.46-1.77) | 0.7690 | 0.9530 | 0.1430 |
| CD8+Ki67+ | 0.94 (0.44-2.03) | 0.8820 | 0.6750 | 0.1120 |
| CD4+FOXP3+ | 0.68 (0.33-1.40) | 0.2900 | 0.9650 | 0.1340 |
| CD4+Ki67+ | 0.13 (0.03-0.59) | **0.0080** | 0.2840 | 0.0911 |
| SYN+ | 0.56 (0.30-1.05) | 0.0722 | 0.7810 | 0.1690 |
| SYN+ Ki67+ | 0.19 (0.04-0.82) | **0.0263** | 0.7390 | 0.1780 |
| TCRδ2+ | 1.80 (0.85-3.79) | 0.1240 | 0.1330 | 0.0548 |
| CD68+ | 0.69 (0.33-1.45) | 0.3280 | 0.7080 | 0.1150 |
| CD56+SYN- | 1.58 (0.83-3.03) | 0.1660 | 0.0234 | 0.0342 |
| CD68+CD163-HLA-DR+ | 1.54 (0.64-3.73) | 0.3340 | 0.1430 | 0.0668 |
| CD68+CD163+HLA-DR- | 0.78 (0.41-1.48) | 0.4510 | 0.7490 | 0.1230 |
| CD68+CD163+HLA-DR+ | 2.56 (1.00-6.56) | 0.0502 | 0.6090 | 0.1270 |
| CD8+ LAG-3+ | 0.46 (0.16-1.33) | 0.1500 | 0.0106 | 0.0348 |
| FOXP3+ LAG-3+ | 1.45 (0.68-3.08) | 0.3370 | 0.2500 | 0.1160 |
| CD56+LAG-3+ | 0.43 (0.10-1.87) | 0.2620 | 0.9140 | 0.1210 |
| SYN+LAG-3+ | 0.61 (0.26-1.44) | 0.2610 | 0.1710 | 0.0976 |
| CD20+ | 2.30 (1.20-4.39) | **0.0120** | 0.5410 | 0.1800 |
| CD3+ | 1.92 (0.67-5.51) | 0.2280 | 0.6790 | 0.1480 |
| CD3+ Ki67+ | 0.56 (0.26-1.20) | 0.1370 | 0.9510 | 0.1390 |
| PD-L1+ | 0.3 (0.11-0.80) | **0.0162** | 0.1070 | 0.1430 |
| CTLA-4+ | 0 (0-Inf) | 0.9970 | 1.0000 | 0.2000 |
| TIM-3+ | 0.57 (0.29-1.12) | 0.1040 | 0.9830 | 0.1480 |
| PD-1+ | 0.58 (0.26-1.29) | 0.1840 | 0.7700 | 0.1690 |
| CD4+ TIM-3+ | 0.78 (0.38-1.60) | 0.4930 | 0.2540 | 0.0748 |
| CD8+ TIM-3+ | 0.79 (0.24-2.62) | 0.7020 | 0.1250 | 0.0643 |
| SYN+ TIM-3+ | 0.33 (0.10-1.08) | 0.0671 | 0.4080 | 0.1180 |
| CD4+ PD-1+ | 0.32 (0.08-1.35) | 0.1210 | 0.0955 | 0.0460 |
| CD8+ PD-1+ | 0.49 (0.12-2.07) | 0.3330 | 0.5190 | 0.1410 |
| SYN+ PD-L1+ | 0 (0-Inf) | 0.9970 | 1.0000 | 0.1590 |

**Supplementary Table 11. Multivariate Cox proportional hazards regression (*high vs low*) model for PFS in MB (n=249)**

| **Variable** | **HR (95%CI)** | ***p value*** | **Ph *p* biomarker** | **Ph *p* global** |
| --- | --- | --- | --- | --- |
| CD4+ | 3.47 (0.82-14.72) | 0.0921 | 0.9450 | 0.7540 |
| CD8+ | 0.41 (0.10-1.70) | 0.2170 | 0.6410 | 0.7010 |
| CD8+Ki67+ | 0.79 (0.42-1.46) | 0.4430 | 0.0132 | 0.2220 |
| CD4+FOXP3+ | 1.53 (0.70-3.32) | 0.2850 | 0.5080 | 0.6950 |
| CD4+Ki67+ | 0.65 (0.27-1.53) | 0.3220 | 0.6220 | 0.5590 |
| SYN+ | 0.44 (0.25-0.80) | **0.0075** | 0.5630 | 0.6560 |
| SYN+ Ki67+ | 0.67 (0.29-1.55) | 0.3520 | 0.7640 | 0.6910 |
| TCRδ2+ | 0.67 (0.33-1.35) | 0.2660 | 0.2660 | 0.6710 |
| CD68+ | 0.72 (0.40-1.30) | 0.2760 | 0.7570 | 0.6680 |
| CD56+SYN- | 2.83 (1.31-6.11) | **0.0082** | 0.6130 | 0.7930 |
| CD68+CD163-HLA-DR+ | 0.68 (0.27-1.73) | 0.4180 | 0.3330 | 0.6030 |
| CD68+CD163+HLA-DR- | 2.84 (1.38-5.84) | **0.0044** | 0.1620 | 0.4650 |
| CD68+CD163+HLA-DR+ | 2.04 (0.87-4.79) | 0.1030 | 0.9080 | 0.7200 |
| CD8+ LAG-3+ | 1.63 (0.75-3.54) | 0.2180 | 0.2290 | 0.6630 |
| FOXP3+ LAG-3+ | 0.75 (0.31-1.82) | 0.5290 | 0.2840 | 0.6600 |
| CD56+LAG-3+ | 0.69 (0.33-1.44) | 0.3180 | 0.2170 | 0.6190 |
| SYN+LAG-3+ | 0.56 (0.26-1.25) | 0.1570 | 0.3020 | 0.6400 |
| CD20+ | 2.38 (1.32-4.32) | **0.0041** | 0.9120 | 0.7010 |
| CD3+ | 0.15 (0.02-1.08) | 0.0597 | 0.2790 | 0.6260 |
| CD3+ Ki67+ | 0.26 (0.04-1.91) | 0.1860 | 0.6930 | 0.7470 |
| PD-L1+ | 0.63 (0.33-1.23) | 0.1750 | 0.6460 | 0.7340 |
| CTLA-4+ | 2.10 (1.11-3.95) | **0.0218** | 0.5970 | 0.6770 |
| TIM-3+ | 0.35 (0.19-0.65) | **0.0008** | 0.9640 | 0.6770 |
| PD-1+ | 1.64 (0.72-3.71) | 0.2380 | 0.6550 | 0.7170 |
| CD4+ TIM-3+ | 0.75 (0.38-1.44) | 0.3830 | 0.8850 | 0.7040 |
| CD8+ TIM-3+ | 0.65 (0.33-1.25) | 0.1980 | 0.2500 | 0.6390 |
| SYN+ TIM-3+ | 0.52 (0.27-1.00) | 0.0516 | 0.1800 | 0.6420 |
| CD4+ PD-1+ | 0.23 (0.06-0.98) | **0.0467** | 0.8840 | 0.6960 |
| CD8+ PD-1+ | 0.88 (0.31-2.51) | 0.8160 | 0.9270 | 0.7290 |
| SYN+ PD-L1+ | 0.45 (0.24-0.85) | **0.0134** | 0.0774 | 0.5310 |

**Supplementary Table 12.** **External clinical information used for verification**

| **Characteristic** | **Group3**  **(N=103)** | **Group4**  **(N=202)** | **SHH**  **(N=98)** | **Overall**  **(N=403)** |
| --- | --- | --- | --- | --- |
| **Age** |  |  |  |  |
| Mean (SD) | 6.11 (3.15) | 8.51 (3.52) | 5.72 (5.26) | 7.22 (4.14) |
| Median [Min, Max] | 5.50 [1.25, 17.0] | 8.00 [1.00, 18.0] | 4.02 [0.240, 18.0] | 7.00 [0.240, 18.0] |
| **Sex** |  |  |  |  |
| F | 38 (36.893%) | 55 (27.228%) | 42 (42.857%) | 135 (33.499%) |
| M | 64 (62.136%) | 146 (72.277%) | 55 (56.122%) | 265 (65.756%) |
| Missing | 1 (0.971%) | 1 (0.495%) | 1 (1.020%) | 3 (0.744%) |
| **M_status** |  |  |  |  |
| M+ | 40 (38.835%) | 82 (40.594%) | 16 (16.327%) | 138 (34.243%) |
| M0 | 63 (61.165%) | 120 (59.406%) | 82 (83.673%) | 265 (65.757%) |
| **Histology** |  |  |  |  |
| Classic | 66 (64.078%) | 167 (82.673%) | 36 (36.735%) | 269 (66.749%) |
| Desmoplastic | 9 (8.738%) | 14 (6.931%) | 45 (45.918%) | 68 (16.873%) |
| LCA | 27 (26.214%) | 15 (7.426%) | 10 (10.204%) | 52 (12.903%) |
| MBEN | 1 (0.971%) | 6 (2.970%) | 7 (7.143%) | 14 (3.474%) |
| **OS** |  |  |  |  |
| Mean (SD) | 50.3 (40.1) | 63.2 (45.2) | 59.3 (40.8) | 58.9 (43.1) |
| Median [Min, Max] | 37.6 [0.260, 173] | 51.2 [1.00, 228] | 47.6 [1.00, 187] | 48.0 [0.260, 228] |
| Missing | 1 (0.971%) | 7 (3.465%) | 2 (2.041%) | 10 (2.481%) |
| **PFS** |  |  |  |  |
| Mean (SD) | 41.3 (41.4) | 53.9 (45.1) | 51.8 (41.3) | 50.0 (43.3) |
| Median [Min, Max] | 24.0 [0.620, 167] | 40.0 [0, 155] | 39.0 [1.43, 187] | 32.0 [0, 187] |
| Missing | 55 (53.398%) | 115 (56.931%) | 49 (50.000%) | 219 (54.342%) |

**External biomarker data (All):**806 patients, 54266 biomarkers.

**External clinical information used for verification (Age ≤18):** 409 patients, 10 variables.

Exclude NOS (not otherwise specified; 1 patient) + WNT (no progression, no death, 5 patients): 403 patients.

**Supplementary Table 13. Multivariate Cox proportional hazards regression model (*high vs low*) for OS in external data (n=381)**

| **Variable** | **HR (95%CI)** | ***p value*** | **Ph *p* biomarker** | **Ph *p* global** |
| --- | --- | --- | --- | --- |
| CD4 | 0.99 (0.53-1.82) | 0.9620 | 0.4240 | 0.0612 |
| CD8A | 0.51 (0.31-0.84) | **0.0076** | 0.3030 | 0.0440 |
| GZMB | 0.76 (0.51-1.13) | 0.1700 | 0.1350 | 0.0125 |
| FOXP3 | 1.03 (0.57-1.84) | 0.9300 | 0.1970 | 0.0607 |
| SYN | 0.69 (0.34-1.38) | 0.2920 | 0.2380 | 0.0467 |
| TRDC | 0.71 (0.46-1.10) | 0.1300 | 0.2060 | 0.0137 |
| CD68 | 1.44 (0.88-2.36) | 0.1480 | 0.8310 | 0.0558 |
| NCAM1 | 0.95 (0.60-1.51) | 0.8280 | 0.9500 | 0.0662 |
| CD86 | 0.86 (0.57-1.29) | 0.4630 | 0.3860 | 0.0583 |
| CD163 | 0.74 (0.30-1.87) | 0.5280 | 0.7500 | 0.0596 |
| MS4A1 | 2.21 (1.34, 3.65) | **0.0020** | 0.3060 | 0.0656 |
| CD3G | 0.72 (0.45-1.15) | 0.1680 | 0.6670 | 0.0591 |
| CD274 | 0.50 (0.33-0.76) | **0.0012** | 0.0142 | 0.0180 |
| CTLA4 | 0.87 (0.53-1.44) | 0.5940 | 0.8580 | 0.0692 |
| LAG3 | 0.66 (0.42-1.03) | 0.0642 | 0.1450 | 0.0263 |
| TIMP3 | 0.75 (0.48-1.17) | 0.2030 | 0.9790 | 0.0680 |
| PDCD1 | 0.91 (0.63-1.33) | 0.6390 | 0.7780 | 0.0667 |

**Supplementary Table 14. Multivariate Cox proportional hazards regression model (*high vs low*) for PFS in external data (n=262)**

| **Variable** | **HR (95%CI)** | ***p value*** | **Ph *p* biomarker** | **Ph *p* global** |
| --- | --- | --- | --- | --- |
| CD4 | 1.89 (1.12-3.20) | **0.0170** | 0.7120 | 0.3210 |
| CD8A | 0.49 (0.30-0.81) | **0.0049** | 0.6540 | 0.2340 |
| GZMB | 0.56 (0.31-0.99) | **0.0475** | 0.7070 | 0.2680 |
| FOXP3 | 1.77 (1.04-3.03) | **0.0367** | 0.7630 | 0.2760 |
| SYN | 0.61 (0.36-1.06) | 0.0807 | 0.0456 | 0.0739 |
| TRDC | 0.60 (0.36-1.01) | 0.0538 | 0.3340 | 0.2730 |
| CD68 | 1.85 (0.88-3.88) | 0.1040 | 0.7450 | 0.2680 |
| NCAM1 | 0.86 (0.48-1.52) | 0.5980 | 0.1660 | 0.1930 |
| CD86 | 1.89 (1.10-3.25) | **0.0211** | 0.8470 | 0.2760 |
| CD163 | 0.58 (0.32-1.08) | 0.0855 | 0.3920 | 0.1670 |
| MS4A1 | 2.15 (1.28-3.61) | **0.0038** | 0.7030 | 0.3290 |
| CD3G | 0.73 (0.43-1.26) | 0.2570 | 0.2630 | 0.0888 |
| CD274 | 0.82 (0.48-1.41) | 0.4690 | 0.1060 | 0.1480 |
| CTLA4 | 1.83 (1.10-3.02) | **0.0189** | 0.9620 | 0.2620 |
| LAG3 | 0.57 (0.33-0.97) | **0.0395** | 0.8290 | 0.3260 |
| TIMP3 | 1.51 (0.81-2.80) | 0.1940 | 0.2600 | 0.2420 |
| PDCD1 | 0.78 (0.48-1.26) | 0.3090 | 0.8330 | 0.2370 |

**Supplementary Table 15. The correspondence between internal data and external data markers.**

| **Internal date** | **External data** |
| --- | --- |
| CD4+ | CD4 |
| CD8+ | CD8A |
| CD8+GZMB+ | GZMB |
| CD4+FOXP3+ | FOXP3 |
| SYN+ | SYN |
| TCR2+ | TRDC |
| CD68+ | CD68 |
| CD56+SYN- | NCAM1 |
| CD68+CD163-HLA-DR+ (M1) | CD86 |
| CD68+CD163+HLA-DR- (M2) | CD163 |
| CD20+ | MS4A1 |
| CD3+ | CD3G |
| PD-L1+ | CD274 |
| CTLA4+ | CTLA4 |
| LAG3+ | LAG3 |
| TIM-3+ | TIMP3 |
| PD-1+ | PDCD1 |
